# Human reproduction is regulated by retrotransposons derived from ancient *Hominidae*-specific viral infections

**DOI:** 10.1038/s41467-022-28105-1

**Published:** 2022-01-24

**Authors:** Xinyu Xiang, Yu Tao, Jonathan DiRusso, Fei-Man Hsu, Jinchun Zhang, Ziwei Xue, Julien Pontis, Didier Trono, Wanlu Liu, Amander T. Clark

**Affiliations:** 1 Zhejiang University-University of Edinburgh Institute (ZJU-UoE Institute), Zhejiang University School of Medicine, International Campus, Zhejiang University, 718 East Haizhou Rd., Haining, 314400 China; 2 Department of Molecular Cell and Developmental Biology, University of California, Los Angeles, Los Angeles, CA 90095 USA; 3 Molecular Biology Institute, University of California, Los Angeles, Los Angeles, CA 90095 USA; 4 School of Life Sciences, Ecole Polytechnique Fe ´de ´rale de Lausanne (EPFL), 1015 Lausanne, Switzerland; 5 Department of Orthopedic Surgery of the Second Affiliated Hospital of Zhejiang University School of Medicine, Zhejiang University, Hangzhou, 310029 China; 6 Dr. Li Dak Sum & Yip Yio Chin Center for Stem Cell and Regenerative Medicine, Zhejiang University, Hangzhou, Zhejiang 310058 China; 7 Alibaba-Zhejiang University Joint Research Center of Future DigitalHealthcare, Zhejiang University, Hangzhou, Zhejiang 310058 China; 8 Eli and Edythe Broad Center of Regenerative Medicine and Stem Cell Research, University of California, Los Angeles, Los Angeles, CA 90095 USA; 9 Jonsson Comprehensive Cancer Center, University of California, Los Angeles, Los Angeles, CA 90095 USA

**Keywords:** Oogenesis, Spermatogenesis, Epigenetics

## Abstract

Germ cells are essential to pass DNA from one generation to the next. In human reproduction, germ cell development begins with the specification of primordial germ cells (PGCs) and a failure to specify PGCs leads to human infertility. Recent studies have revealed that the transcription factor network required for PGC specification has diverged in mammals, and this has a significant impact on our understanding of human reproduction. Here, we reveal that the *Hominidae*-specific Transposable Elements (TEs) LTR5Hs, may serve as TEENhancers (TE Embedded eNhancers) to facilitate PGC specification. LTR5Hs TEENhancers become transcriptionally active during PGC specification both in vivo and in vitro with epigenetic reprogramming leading to increased chromatin accessibility, localized DNA demethylation, enrichment of H3K27ac, and occupation of key hPGC transcription factors. Inactivation of LTR5Hs TEENhancers with KRAB mediated CRISPRi has a significant impact on germ cell specification. In summary, our data reveals the essential role of *Hominidae*-specific LTR5Hs TEENhancers in human germ cell development.

## Abstract (web summary)

The transcription factor network required for primordial germ cell (PGC) specification is known to diverge in mammals. Here the authors show that hominidae-specific transposable element (TE) LTR5Hs becomes transcriptionally active during PGC specification, and LTR5Hs inactivation abrogates human PGC specification

## Introduction

Proper formation of the adult germline is essential for the passage of genetic and epigenetic information from generation to generation. Primordial Germ Cells (PGCs) are specified during early embryonic development and constitute the founder germline cells that ultimately give rise to oocytes and sperm in the adult. As such, failure to specify PGCs leads to certain infertility in adulthood. Given the central importance of PGCs to reproduction, the developmental cues and regulatory milieu governing specification of PGCs has been broadly studied in various animal models^1^. While these models have proven instructive in PGC biology, constraints imposed by ethical and technical limitations have rendered the precise mechanisms governing human (h) PGC (hPGC) specification in vivo unclear.

Human PGCs originate from peri-implantation progenitors at day 11-12 (D11-12) post-fertilization just before gastrulation^2^, a time point at which clinical samples are prohibitively rare. Due to the inaccessibility of early hPGC development in vivo, an in vitro system for differentiating hPGC-Like Cells (hPGCLCs) from human pluripotent stem cells (hPSCs) has been established^3,4^. Using this system, both conserved and unique transcriptional networks regulating hPGC specification have been uncovered^3–7^. For instance, NANOG, PRDM1, TFAP2C, and PRDM14 are required for PGC specification and maintenance in both human and mouse embryos^2,8–13^. In contrast, SOX17 is crucial for hPGC specification^3^, but is dispensable in mouse; where instead SOX2 regulates the specification of mouse PGCs^14,15^. In addition to the transcription factors (TFs), differences can also be found in the gene regulatory elements required to specify PGCs, such as the utilization of a naïve enhancer at the *POU5F1* (*OCT4*) locus in hPGCs^16^, whereas this naïve enhancer sequence is not conserved in mouse^17^. Given this, we hypothesized that an additional source of variance in the regulatory networks governing PGC specification could be associated with transposable elements (TEs); repetitive elements which account for around half of the human genome.

Most of the TEs in the human genome are retrotransposons, which propagate through an RNA intermediate. Specifically, retrotransposon sequences are first transcribed as RNA, followed by reverse transcription to DNA before integration of a new copy into the genome^18^. Based on function and structure, retrotransposons are further classified as LINE- (long interspersed nuclear elements), SINE- (short interspersed nuclear elements), LTR- (long terminal repeats), or the *Hominidae*-specific SVA (SINE-VNTR-Alu)-elements^18^. Of particular interest when considering TE contribution to the regulatory landscape of the genome are Endogenous retroviruses (ERVs), a superfamily within the LTR retrotransposon class.

ERVs originate from ancient viruses that infected and integrated into the germline throughout evolution. Most Human ERVs appear to have entered the germline after the new world and old world monkey split^19–23^. Even though LTR retrotransposons occupy ~8% of the human genome, almost all LTR retrotransposon sequences have lost their transposition ability^18^. Nevertheless, recent studies suggest that LTR retrotransposons, especially ERVs, can serve as regulatory sequences that participate in gene regulation networks^24^. In humans, ERV sequences harbor binding sites for OCT4, NANOG, and p53^25,26^. Specifically, ChIP-seq analysis has shown that human ERV elements account for roughly 25% of all bound NANOG and OCT4^25^ and nearly one-third of all p53 binding sites^26^, demonstrating a profound contribution by human ERVs to the human regulatory landscape.

The most recent expansion of human ERVs occurred over the last 5-20 million years in the HERVK (human mouse mammary tumor virus like-2, HML-2) group^27^. Even though HERVK(HML2) elements are also found in old world primates, distinct phylogenetic differences exist between those found in *Hominidae* relative to *Hominoidea*. For example, HERVK(HML2) elements which are found in both monkey and human genomes have LTR5A and LTR5B regulatory sequences, while the most recent *Hominidae*-specific HERVK(HML2) elements harbor the LTR5Hs regulatory sequence^27^. In addition, some HERVK(HML2) TEs contain intact open reading frames that can code for viral proteins^28^, with LTR5Hs-regulated HERVK(HML2) provirus expression proposed to be a property of naïve human embryonic stem cells (hESCs)^29^. Grow and colleagues further hypothesize that expression of full-length LTR5Hs-regulated HERVK(HML2) proviruses may confer a critical immunoprotective effect in the human pre-implantation embryo by stimulating IFITM-1, a viral restriction factor, potentially protecting against HERVK(HML-2)-like retrovirus re-infection^29^.

In addition to the production of viral particles, it is also known that many HERVK/LTR5Hs-, SVA-, and HERVH/LTR7- elements in the genome are accessible and marked by H3K27ac in human pluripotent cells, suggesting that they also serve a potential gene regulatory function associated with pluripotency^30^. Consistent with this, key pluripotency factors of the KLF family bind to and activate evolutionarily young TE Embedded eNhancers (TEENhancers) found in LTR5Hs and SVA elements to facilitate human embryonic genome activation^30^. Thus, evolutionarily young *Hominidae*-specific TEs have extensively shaped the regulatory landscape of early embryonic development and this has likely fostered species divergence in the gene regulatory networks that regulate the development of cells in the reproduction system.

Here, we discovered that the *Hominidae*-specific LTR5Hs is expressed in hPGCs in vivo and hPGCLCs in vitro. Increased expression of LTR5Hs in hPGCLCs is associated with a remodeled epigenetic landscape leading to increased chromatin accessibility and localized DNA demethylation. Substantial binding of TFAP2C, NANOG, SOX17, SOX15, and enriched H3K27ac histone marks at LTR5Hs loci suggest a TEENhancer role for these TEs in hPGC specification. Inactivation of LTR5Hs TEENhancers compromises hPGCLC formation and de-regulates germline gene expression. In summary, our results reveal that LTR5Hs TEENhancers are involved in hPGC specification, and thus may cultivate the species specificity in human reproduction.

## Results

### Up-regulation of LTR5Hs transcript abundance in germline lineage

**Fig. 1:**
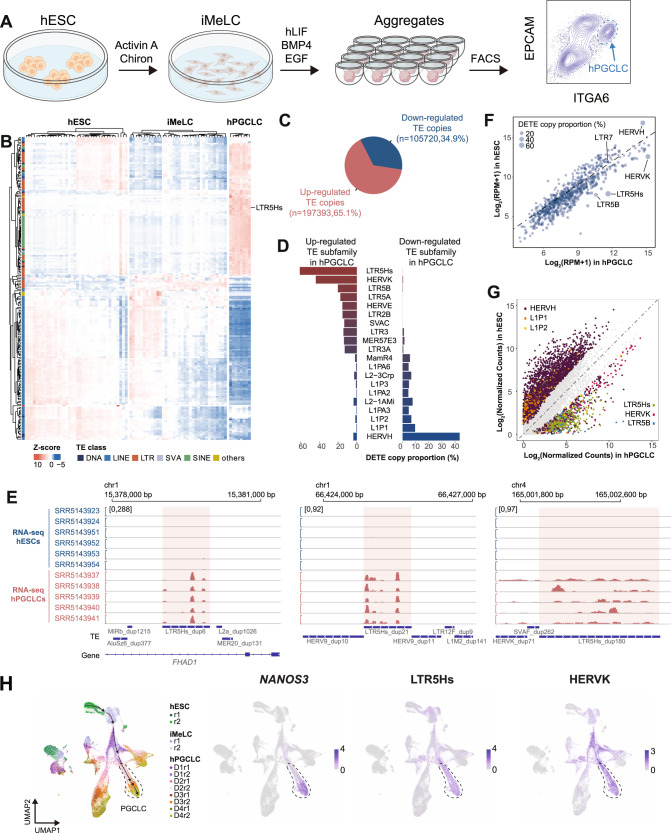
Lineage-specific up-regulation of LTR5Hs in hPGCLC induction. **A** Schematic illustration of hPGCLC in vitro differentiation procedure. **B** Heatmap for the top 200 TE subfamilies with the highest cross-sample variation in hESCs, iMeLCs, and hPGCLCs. The colored bar on the left indicates TE class. **C** Pie chart showing the proportion of up- or down-regulated DETE copies in hPGCLCs compared to hESCs using a cut-off of at least a 4-fold change and FDR <0.05. **D** Top 10 up- or down-regulated TE subfamilies in hPGCLCs. X axis shows DETE copy numbers proportional to the total copy number of a specific TE subfamily. Only TE subfamilies with at least 80 copies and 8 DETE copies are kept for this analysis. **E** Screenshots showing representative hESC and hPGCLC RNA-seq tracks over LTR5Hs integrants. Red shaded rectangle region indicates individual LTR5Hs copies. **F** Scatterplot for aggregated expression level of each TE subfamily in hESCs and hPGCLCs. The size of each dot represents the proportion of DETE copy numbers relative to the total copy number of each TE subfamily. **G** Scatterplot of the expression of individual TE copies belonging to the top three up- or down-regulated DETE subfamilies. Gray dots represent TE copies which are not differentially expressed. **H** UMAP of scRNA-seq dataset for two replicates (r) of UCLA2 hESCs, iMeLCs, and D1 to D4 hPGCLCs (left), representative expression pattern for *NANOS3*, LTR5Hs, and HERVK. Differentiation trajectory of hPGCLCs is denoted by arrows, hPGCLC population is indicated by dashed line. DETE analysis for this figure is analyzed by the STAR + featureCounts+DESeq2 method. Source data underlying **B**, **D**, and **F** are provided as a Source Data file.

In order to characterize dynamically expressed TE subfamilies during germ cell specification, we analyzed the RNA-seq data sets previously published from our lab^16^, including day 4 (D4) human PGC-like cells (hPGCLCs) differentiated from primed state hESCs through incipient mesoderm like cells (iMeLC) (Fig. 1A and Supplementary Data 1), an intermediate cell type between primed hESCs and hPGCLCs^4^. The D4 hPGCLCs are transcriptionally equivalent to early primate PGCs between D11-D21 post-fertilization^2^. To overview the expression pattern of TEs during hPGCLC induction, the top 200 TE subfamilies with the highest cross-sample variation were visualized (Fig. 1B). In general, we observed dynamic TE expression patterns during hPGCLC specification, with LTR5Hs being one of the top highly expressed TE subfamilies in hPGCLCs (Fig. 1B).

Since TEs are repetitive sequences in the genome, TE-derived RNA-seq reads are hard to quantify. To further identify TE subfamilies specific to hPGCLCs, we called differential expressed TE copies (DETE) in hPGCLCs compared with hESCs using a variety of methods recommended for TE quantification^31–35^. Briefly, RNA-seq reads were aligned to the reference genome with STAR or SQuIRE^32,36^ (Supplementary Fig. 1A). Then, TE-derived RNA-seq reads over individual TE copies were quantified with featureCounts, SQuIRE, Telescope, or TEtranscripts^32–35^ followed by DETE calling with DESeq2^37^ (Supplementary Fig. 1A and Supplementary Data 2–5). Using a four-fold difference and false discovery rate (FDR) <0.05 as a cut-off, we identified more up-regulated DETE copies in hPGCLCs compared to hESCs (65.1% for featureCounts; 66.1% for Telescope; 71.4% for TEtranscripts) except for SQuIRE (48.7%) (Fig. 1C and Supplementary Fig. 1B). Since different TE subfamilies possess variable copy numbers, we reasoned large absolute DETE copy numbers may be due to the high total copy number for certain TE subfamilies. Therefore, to reveal TE subfamilies that are most dynamically expressed in hPGCLCs, we calculated the DETE copy numbers proportional to the total copy numbers for a specific TE subfamily and plotted the top 10 up- or down-regulated TE subfamilies in hPGCLCs (Fig. 1D and Supplementary Fig. 1C).

With different methods, we consistently observed primate- and *Hominidae*-specific TEs including LTR5Hs/HERVK as top up-regulated, and HERVH as top down-regulated TE subfamilies in hPGCLCs (Fig. 1D, E and Supplementary Fig. 1C). We next analyzed the aggregated transcript abundance for each TE subfamilies and obtained similar conclusions (Fig. 1F and Supplementary Fig. 2A). To better display the transcript abundance dynamics for DETE subfamilies, we also plotted the individual DETE copies for the top 3 up- or down-regulated DETE subfamilies, confirming the up-regulation of LTR5Hs/HERVK in hPGCLCs (Fig. 1G and Supplementary Fig. 2B).

LTR5Hs serves as the regulatory elements for HERVK, while LTR7 serves as the regulatory elements for HERVH^29,38^. We observed up-regulation of both LTR5Hs and HERVK with hPGCLC induction and down-regulation of HERVH, while LTR7 expression levels were unchanged with hPGCLC induction (Fig. 1D, F-G and Supplementary Fig. 1C and 2A–C). As recombination of ERVs leads to the formation of solo-LTRs in the genome^39^, we wanted to evaluate the transcript abundance of provirus-associated LTR5Hs or LTR7 compared to solo-LTR5Hs or solo-LTR7. To do so, we classified LTR5Hs and LTR7 further into HERVK-LTR5Hs, solo-LTR5Hs, HERVH-LTR7, and solo-LTR7. Transcript abundance analysis indicated significant up-regulation of both HERVK-LTR5Hs and solo-LTR5Hs in hPGCLCs (Supplementary Fig. 2D). However, expression levels of HERVH-LTR7 and solo-LTR7 showed no significant changes between hESCs and hPGCLCs (Supplementary Fig. 2D). Our observations suggested that the down-regulation of HERVH in hPGCLCs is uncoupled from expression changes at LTR7.

To investigate the expressions of TEs during hPGCLC induction with single-cell resolution, we re-analyzed the 10X Genomics single-cell RNA-seq data published by our lab^2^. Using *NANOS3* as a marker for hPGCLCs, we clearly identified the up-regulation of LTR5Hs, HERVK and down-regulation of HERVH with differentiation of hPGCLCs, while the expression of other selective TE subfamilies were either at background levels or not specific to hPGCLCs (Fig. 1H and Supplementary Fig. 3). For additional interrogation of TEs expressed by hPGCs in vivo or hPGCLCs in vitro the following searchable website has been created and is freely available at https://labw.org/germlineTE/.

Human in vivo PGCs start to emerge around embryonic D11-D12^2^. To determine whether newly specified hPGCs in vivo express LTR5Hs, we re-analyzed the scRNA-seq (SMART-Seq) data from two Carnegie Stage 7 (CS7) embryos corresponding to embryonic D15 and D17 post-fertilization^40^. Seven hPGCs were annotated by Tyser et al. in this data set, and four sets of seven other randomly chosen cells were annotated as epiblast, primitive streak, emergent mesoderm, and advanced mesoderm were included in our analysis of selected TEs (Supplementary Fig. 4A). Using this single-cell RNA-Seq data we showed that LTR5Hs and HERVK are up-regulated in hPGCs in vivo^40^.

We also investigated whether up-regulation of LTR5Hs was specific to hPGCLCs during in vitro somatic cell differentiation by examining the expression of LTR5Hs in RNA-seq datasets from in vitro multilineage differentiation from primed hESCs^41^. This analysis showed that LTR5Hs and HERVK were not enriched during the differentiation of hESCs into mesenchymal stem cell (MSC), neural progenitor cell (NPC), trophoblast-like cell (TBL), and mesendoderm (ME) (Supplementary Fig. 4B). In contrast, and consistent with previous findings, LTR7 and HERVH showed enriched expression in primed hESCs and ME relative to the other cell types^41^ (Supplementary Fig. 4B).

Overall, our in-depth analysis of RNA-seq and scRNA-seq data sets during hPGCLC induction from hESCs, scRNA-seq data from in vivo CS7 PGCs, and RNA-seq data sets from hESC multilineage differentiation collectively showed LTR5Hs is uniquely up-regulated with hPGCLC induction in vitro and is expressed by hPGCs in vivo.

### Increased chromatin accessibility of LTR5Hs in hPGCLCs

**Fig. 2:**
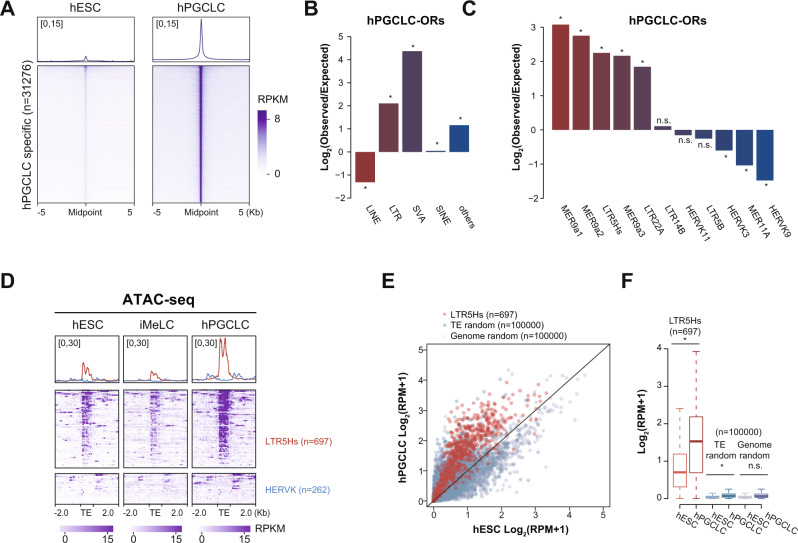
Increased chromatin accessibility over LTR5Hs with hPGCLC induction. **A** Heatmap and metaplot for ATAC-seq signals over hPGCLC-ORs (*n* = 31276). **B**, **C** Enrichment of TE classes (**B**), and TE subfamilies within the ERVK family (**C**) for hPGCLC-ORs over random shuffled regions with comparable genomic distributions (*p-value < 0.05, binomial test; n.s. = not significant). **D** Heatmap and metaplot of ATAC-seq signals over all LTR5Hs (*n* = 697) and HERVK copies (*n* = 262) in hESCs, iMeLCs, and hPGCLCs. **E**, **F** Scatterplot (**E**) and boxplot (**F**) of ATAC-seq signals over LTR5Hs, randomly shuffled TE and genomic regions in hESCs and hPGCLCs (*p-value < 0.05, Welch Two Sample *t*-test; n.s. = not significant). In **F** the middle line represents the median; boxes represent the 25th (bottom) and 75th (top) percentiles; and whiskers represent the minimum and maximum points within 1.5× the interquartile range. Source data underlying **B**, **C**, and **F** are provided as a Source Data file.

Given the potential enhancer role of TEs in regulating gene expression^30,42^, we next evaluated changes in chromatin accessibilities during hPGCLC induction with our previously published Assay for Transposase-Accessible Chromatin sequencing (ATAC-seq) data^16^. Comparing ATAC-seq data of primed-state hESCs, iMeLCs, and hPGCLCs, we identified 31,276 and 90,201 ATAC-seq peaks that become specifically more accessible in hPGCLCs (referred as hPGCLC open regions, hPGCLC-ORs) and hESCs (referred as hESC open regions, hESC-ORs), respectively (Fig. 2A, Supplementary Fig. 5A, and Supplementary Data 6).

To uncover specific TE subfamilies enriched in the hPGCLC-ORs and hESC-ORs, we annotated the genomic distribution of those regions and investigated their enrichment over TE regions. As TEs are not randomly distributed across the genome, we generated randomly shuffled regions as controls by adjusting the relative proportion of genomic distribution comparable to hPGCLC-ORs or hESC-ORs^43^ (Supplementary Fig. 5B). Compared with control regions, our analysis revealed that LTR- and SVA-classes were significantly enriched in both hPGCLC- and hESC-ORs (Fig. 2B and Supplementary Fig. 5C). Further analysis of LTR-class containing open regions showed that ERVK was the top enriched LTR family in both hPGCLC- and hESC-ORs (Supplementary Fig. 5D, E). Within the ERVK family, the enriched TE subfamilies diverged between hPGCLC-ORs and hESC-ORs. Interestingly, MER9a1, MER9a2, and LTR5Hs were ERVK subfamilies that were significantly enriched in hPGCLC-ORs, while LTR22A, MER11B, and LTR22C2 were significantly enriched in hESC-ORs (Fig. 2C and Supplementary Fig. 5F). In addition to the LTR family, we also detected enrichment of SVA family members including SVAC and SVAD in hPGCLC-ORs, and enrichment of SVAB and SVAA in hESC-ORs (Supplementary Fig. 5G, H).

Analysis for ATAC-seq signals over LTR5Hs further confirmed the increased chromatin accessibility over LTR5Hs in hPGCLCs, while the chromatin landscape of HERVK was not accessible in hPGCLCs (Fig. 2D). As a contrast, LTR7 showed comparable chromatin accessibility in hESCs, iMeLCs, and hPGCLCs, while HERVH was not accessible in any of the cell types (Supplementary Fig. 5I). We next quantified the chromatin accessibility of LTR5Hs in hPGCLCs. By comparing to 100,000 randomly chosen TE copies or genomic regions, we observed that the majority of LTR5Hs loci became more open with hPGCLC induction, while we observed no dramatic changes for control regions (Fig. 2E, F).

### Localized hypomethylation over LTR5Hs in hPGCLCs

**Fig. 3:**
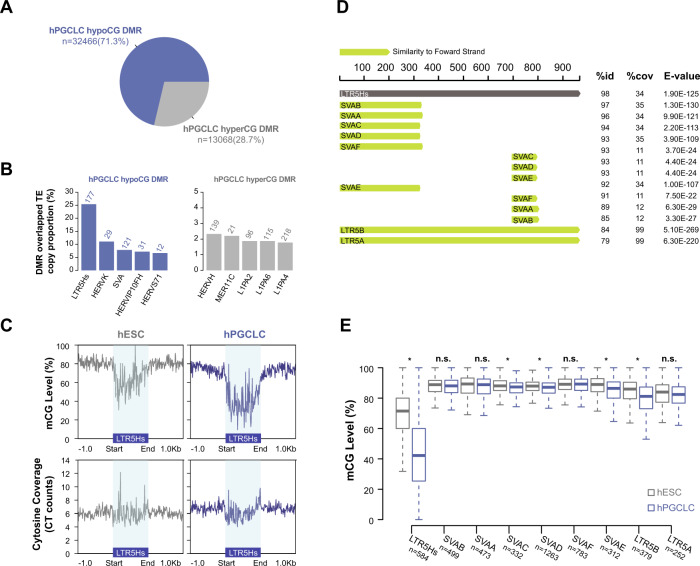
Localized DNA demethylation over LTR5Hs in hPGCLCs. **A** Percentage of hypoCG and hyperCG DMRs in hPGCLCs compared to hESCs. **B** Bar plot showing enrichment of TE subfamilies in hyperCG or hypoCG DMRs as a proportion of total TE copy number. **C** Metaplot of aggregate CG methylation level (top) and cytosine coverage (bottom) over LTR5Hs in hESCs and hPGCLCs. Blue shaded rectangle region indicates annotated LTR5Hs regions. **D** The consensus sequence similarity of LTR5Hs and related TE clades from Dfam^45^. Percent identity between the entry consensus sequences (%id), percent shared coverage (%cov) and match e-value (E-value) are displayed on the right. **E** Boxplot of CG methylation level over LTR5Hs and related TE clades in hESCs and hPGCLCs. Only TE subfamilies with a copy number >100 are included in the plot. *p-value < 0.05, Welch Two Sample *t*-test; n.s. represents not significant. The middle line represents the median; boxes represent the 25th (bottom) and 75th (top) percentiles; and whisker bars represent the minimum and maximum points within the 1.5× interquartile range. Source data underlying **E** is provided as a Source Data file and originates from *n* = 2 biological replicates (separate experiments) of hESCs and hPGCLCs generated from the UCLA2 hESC line.

Considering the chromatin accessibility changes over LTR5Hs, we next examined the DNA methylation landscape of LTR5Hs in hPGCLCs. Our previous study suggested there was no obvious genome-wide DNA demethylation in hPGCLCs compared to hESCs^44^. Consistent with our previous conclusion, re-analysis of our hESC and hPGCLC D4 whole Genome Bisulfite Sequencing (WGBS) data showed comparable average CG methylation in hESCs and hPGCLCs (Supplementary Fig. 6A). However, differential methylated region (DMR) analysis identified 32466 hypomethylated CG (hypoCG, 71.3%) and 13068 hypermethylated CG (hyperCG, 28.7%) DMRs in hPGCLCs compared to primed hESCs (Fig. 3A and Supplementary Data 7). Among those DMRs, we observed LTR5Hs as the top TE subfamily that overlapped with hPGCLC hypoCG DMRs, and HERVH as the top TE subfamily that overlapped with the hPGCLC hyperCG DMRs (Fig. 3B).

Metaplots of CG methylation levels over LTR5Hs revealed CG demethylation across the whole LTR5Hs sequences (Fig. 3C). To rule out mappability issues in highly repetitive sequences, we also examined the cytosine coverages over LTR5Hs and detected comparable mappability within the LTR5Hs regions compared to the flanking genomic sequences (Fig. 3C). SVAD and LTR5Hs share common sequences, and both contribute to maintenance of the transcriptional regulatory network in naïve hESCs^30^ (Fig. 3D and Supplementary Fig. 6B). To investigate whether SVAD was also demethylated in hPGCLCs, we plotted the CG methylation level over SVAD and detected modest demethylation close to the 3′ end of SVAD (Supplementary Fig. 6B). We reasoned this modest demethylation on SVAD was likely due to sequence conservation between LTR5Hs and this region of the SVAD. To test this hypothesis, we next focused on LTR5Hs and related TE clades that share the most sequence similarities with LTR5Hs: SVAB, SVAA, SVAC, SVAD, SVAF, SVAE, LTR5B, and LTR5A, obtained from the Dfam database^45^ (Fig. 3D). Of this clade, only LTR5Hs displayed extreme CG demethylation during hPGCLC induction, while none of other related TE clades showed this trend (Fig. 3E and Supplementary Fig. 6D). This result suggested that the localized demethylation at LTR5Hs is specific to LTR5Hs itself, rather than to the LTR5Hs related sequences in SVA.

Based on our observations of RNA expression, chromatin accessibility, and localized demethylation of LTR5Hs in hPGCLCs, we thus hypothesized that the epigenetic activity of LTR5Hs might mediate a human-specific epigenetic landscape for hPGC specification.

### LTR5Hs may serve as germ cell-specific TEENhancers

A previous study on evolutionary young TEs in human early embryogenesis suggested that LTR5Hs and SVAD elements may serve as TEENhancers, which are involved in species-specific transcriptional networks^30^. To explore whether LTR5Hs functions as TEENhancers in hPGCLC induction, we inspected the binding profiles of key TFs as well as the enhancer histone mark, H3K27ac at LTR5Hs.

Previous studies have shown that Transcription Factor AP-2 (Activating enhancer-binding Protein 2) Gamma (TFAP2C), a TF from the AP2 family, is required for hPGCLC induction^3,16^. SRY-box (Sex-determining Region Y box) TFs SOX17, SOX15 and its downstream target ETV5 have also been reported as critical factors for hPGCLC induction and maintenance^3,7,46^. In addition, homeobox protein NANOG has been proposed as an indispensable pluripotency factor in PGC fate determination^8,47^. Motif analysis of hPGCLC-ORs which overlapped with LTR5Hs showed enrichment of known factors critical for PGC biology, including ets-ebox, AP2, and Sox family (Supplementary Fig. 7A). These results were consistent with reports that the Oct4:Sox17, AP-2 Gamma, and Sox15 motifs were highly enriched in hPGCLC-ORs^16^. To examine the binding of TFs at LTR5Hs in hESCs and hPGCLCs, we evaluated our previous published TFAP2C ChIP-seq (Chromatin Immunoprecipitation followed by sequencing)^2^, previously published H3K27ac ChIP-seq data^2,48^, as well as published SOX15 CUT&Tag-seq (Cleavage Under Targets and Tagmentation)^7^. In addition, we performed ChIP-seq of NANOG and SOX17 in hESCs and hPGCLCs.

**Fig. 4:**
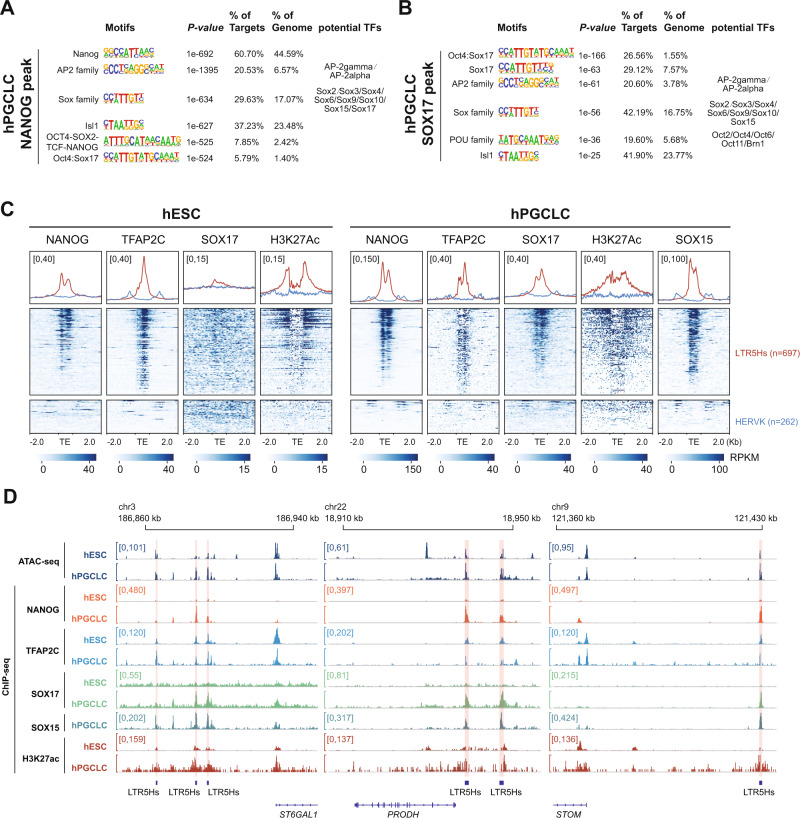
LTR5Hs may serve as TEENhancers in hPGCLCs. **A**, **B** Top enriched motifs within NANOG (**A**) and SOX17 (**B**) ChIP-seq peaks in hPGCLCs. Hypergeometric test is performed using Homer^77^. **C** Heatmaps and metaplots of NANOG, TFAP2C, SOX17, and H3K27ac ChIP-seq signals in hESCs and hPGCLCs, and SOX15 CUT&Tag-seq signals in hPGCLCs over all LTR5Hs (*n* = 697) and HERVK (*n* = 262). **D** Screenshots for ATAC-seq signals, NANOG, TFAP2C, SOX17, and H3K27ac ChIP-seq signals in hESCs and hPGCLCs, and CUT&Tag-seq signals for SOX15 over LTR5Hs TEENhancers and their potential target genes (*ST6GAL1*, *PRODH*, and *STOM*).

Motif analysis for NANOG and SOX17 ChIP-seq peaks in hPGCLCs validated the quality of our ChIP-seq experiments, with the most enriched motif as Nanog and the Oct4:Sox17 fused motif, respectively (Fig. 4A, B). Interestingly, we detected the enrichment of AP2 family, SOX family, and POU family motifs in both the NANOG and SOX17 ChIP-seq peaks in hPGCLCs (Fig. 4A, B). Therefore, our results implied the existence of an interconnected transcriptional regulatory network in hPGCLCs.

To address this, we next analyzed the binding profiles of key TFs and H3K27ac in hESCs and hPGCLCs over LTR5Hs and HERVK with LTR7 and HERVH used as controls (Fig. 4C, Supplementary Fig. 7B, and Supplementary Data 8). Overall, we observed extensive binding of NANOG (39.7%), TFAP2C (58.7%), and an enrichment of H3K27ac, but no binding of SOX17, over the majority of LTR5Hs copies in undifferentiated hESCs (Fig. 4C and Supplementary Fig. 7C). For LTR7, we observed moderate binding of NANOG (31.0%) and TFAP2C (14.6%), and a slight enrichment of H3K27ac in hESCs (Supplementary Fig. 7B, C). In contrast, with differentiation of hPGCLCs we observed universal binding of NANOG (71.3%), TFAP2C (62.4%), SOX15 (60.4%), and SOX17 (24.0%) as well as the enrichment of H3K27ac at LTR5Hs (Fig. 4C and Supplementary Fig. 7D). The binding of key hPGC TFs, as well as enrichment of H3K27ac at LTR5Hs suggests an enhancer role for LTR5Hs with hPGCLC induction. For instance, a 40-kb distal LTR5Hs has been proposed to act as super-enhancer for naïve pluripotency gene *ST6GAL1*^30,49^. We also observed the extensive binding of NANOG, TFAP2C, SOX17, and SOX15 over the LTR5Hs nearby *ST6GAL1* in hPGCLCs (Fig. 4D). Similar binding patterns were observed for LTR5Hs near the hPGCLC up-regulated genes *PRODH* and *STOM* (Fig. 4D). For LTR7, we observed modest binding of NANOG (31.2%) in hPGCLCs with negligible binding of SOX15 (8.8%), TFAP2C (7.4%), SOX17 (0.5%), or H3K27ac enrichment (Supplementary Fig. 7B, D). No signs of NANOG, TFAP2C, SOX17, SOX15, or H3K27ac enrichment were detected over HERVK or HERVH (Fig. 4C and Supplementary Fig. 7B).

The substantial binding of key hPGCLC key TFs, along with the localized remodeling of the epigenetic landscape, led us to propose that LTR5Hs may serve as a hPGCLC-specific TEENhancer to regulate hPGCLC induction.

### LTR5Hs TEENhancers are essential for hPGCLC Induction

**Fig. 5:**
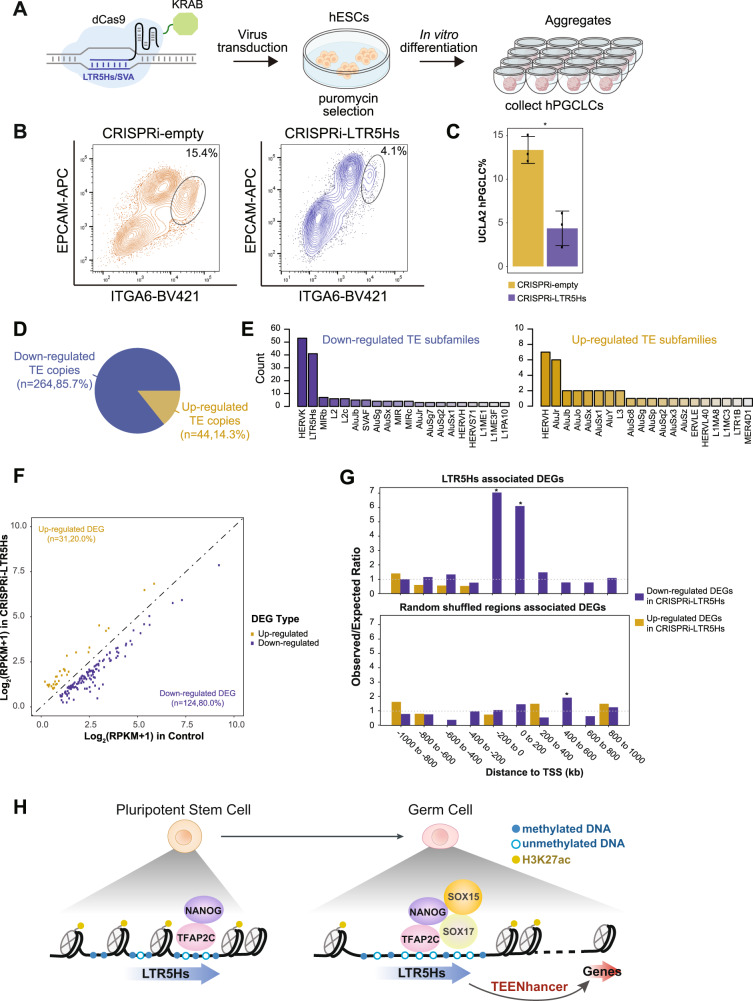
Inactivation of LTR5Hs TEENhancers leads to less hPGCLC formation. **A** Schematic illustration depicting transduction of dCas9-KRAB-gRNAs targeting LTR5Hs in hESCs followed by hESCs differentiation to hPGCLCs. **B** Representative flow cytometry plots for hPGCLC differentiating from CRISPRi-LTR5Hs and CRISPRi-empty in UCLA2 hESC lines. The black circles denote the hPGCLC population as defined by ITGA6/EPCAM double-positive cells. **C** Barplot showing the percentage of hPGCLCs in CRISPRi-empty relative to CRISPRi-LTR5Hs groups in UCLA2 (biological replicates *n* = 3; *p-value = 0.0042; error bars showing mean ± SEM). **D** Pie chart showing up- or down-regulated DETE copies in CRISPRi-LTR5Hs compared with CRISPRi-empty controls, as defined by at least a 4-fold change in expression and FDR <0.05. **E** Barplot of the TE subfamilies with the most up- or down-regulated DETE copies. **F** Scatterplot of the expression level for identified up- or down-regulated DEGs in CRISPRi-LTR5Hs compared with CRISPRi-empty control, using a cut-off of least 1.5-fold change in expression and a FDR of <0.05. **G** RAD analysis for the association between LTR5Hs (upper panel) or random shuffled regions (lower panel) with CRISPRi-LTR5Hs DEGs. *p-value < 0.05, two-sided Welch Two Sample *t*-test. **H** Proposed model for the role of LTR5Hs as TEENhancers in PGC specification. Source data underlying **C** and **G** are provided as a Source Data file.

To evaluate the functional relevance of LTR5Hs TEENhancers in hPGCLC induction, we transduced UCLA2 hESCs lines with lentivirus encoding dCas9-KRAB fusion protein together with validated gRNAs targeting LTR5Hs (referred as CRISPRi-LTR5Hs)^30^ (Fig. 5A). As control, hESCs were transduced with dCas9-KRAB with no gRNAs (referred as CRISPRi-empty). Then, CRISPRi-empty and CRIPSRi-LTR5Hs hESC lines were induced to differentiate into hPGCLCs (Fig. 5A). By tethering KRAB protein to LTR5Hs loci with CRISPRi, the H3K9me3 repressive mark would be induced at targeted loci, thus inactivating LTR5Hs TEENhancers^50^. At day 4 of hPGCLC induction, we quantified the percentage of hPGCLCs using Fluorescence-Activated Cell Sorting (FACS). In the CRISPRi-LTR5Hs lines, we consistently observed a significant reduction in the percentage of hPGCLCs compared to CRISPRi-empty controls (Fig. 5B, C). We further validated our results by repeating the experiments in the UCLA1 hESC line and obtained the same conclusion (Supplementary Fig. 8A). Collectively, our results suggested LTR5Hs TEENhancers are involved in hPGCLC induction.

To uncover potential downstream LTR5Hs TEENhancer-regulated genes involved in hPGCLC biology, we performed RNA-seq of CRISPRi-LTR5Hs and CRISPRi-empty sorted hPGCLCs. Analyzing the DETE copies in CRISPRi-LTR5Hs compared with CRISPRi-empty, we detected 264 (85.7%) down-regulated and 44 (14.3%) up-regulated TE copies, with HERVK and LTR5Hs as TE subfamily with the most down-regulated DETE copies and HERVH with the most up-regulated DETE copies (Fig. 5D, E and Supplementary Data 9).

We then scanned the potential target sites for LTR5Hs gRNAs in the human genome by allowing a maximum of three mismatches with the LTR5Hs targeting guides. In total, we identified 6044 predicted target sites for the two gRNAs used to target LTR5Hs, among which 942 (15.59%) were located on 76.76% of all LTR5Hs copies (Supplementary Fig. 8B and Supplementary Data 10). Consistent with previous findings, SVA family members, especially SVAD, were also predicted to be targeted by the two gRNAs^30^, while few predicted sites targeted to genic regions (Supplementary Fig. 8B). Even though the gRNAs could be targeted to SVAD, we found no evidence for down-regulation of SVAD in CRISPRi-LTR5Hs hPGCLCs (Supplementary Fig. 8C). Using the same gRNAs, Pontis *et al*. observed significant repression of SVAD in CRISPRi-LTR5Hs naïve hESCs^30^ (Supplementary Fig. 8C). This difference between naïve hESCs and hPGCLCs is likely due to the very low SVAD expression levels in hPGCLCs compared to naïve hESCs (Supplementary Fig. 8C). Additionally, as our hPGCLCs are differentiated from primed hESCs in which SVAD elements are not expressed (this study) or adorned with H3K27ac (Pontis et al.^30^), we would not expect interference in hPGCLC induction from off-target SVAD silencing. Overall, we detected significant down-regulation of LTR5Hs, HERVK, and up-regulation of HERVH in CRISPRi-LTR5Hs hPGCLC while no changes in SVAD or LTR7 (Supplementary Fig. 8C, D).

We then analyzed the effect of CRISPRi-LTR5Hs on gene expression. Consistent with the DETE pattern, we detected 124 (80%) of down-regulated DEGs (differential expressed genes) (using 1.5-fold change and FDR <0.05 as cut-off), while only 31 (20%) DEGs were up-regulated in CRISPRi-LTR5Hs compared to control (Fig. 5F and Supplementary Data 11). Considering the mild gene expression changes, we also included a MA plot to control for data normalization during DEG calling (Supplementary Fig. 8E).

To evaluate whether LTR5Hs was significantly associated with the DEGs in CRISPRi-LTR5Hs, we employed RAD (Region Associated DEG) analysis^51^. With this analysis, we discovered that down-regulated DEGs in CRISPRi-LTR5Hs were significantly enriched within 200 kb next to LTR5Hs copies (Fig. 5G and Supplementary Data 12). As a control, no significant association was found between randomly shuffled regions and CRISPRi-LTR5Hs DEGs (Fig. 5G and Supplementary Data 12). Correspondingly, RAD analysis for CRISPRi gRNAs predicted sites associated CRISPRi-LTR5Hs DEGs showed a similar pattern (Supplementary Fig. 8F and Supplementary Data 13).

To rule out the possibility that the decrease in hPGCLC induction in the CRISPRi-LTR5Hs was derived from indirect effects, such as differentiation delay, or loss of pluripotency, we examined the expression of hPGC and pluripotency marker genes (Supplementary Fig. 9A). We detected no obvious changes in expression of these marker genes in CRISPRi-LTR5Hs and control samples, and thus conclude that the reduced induction of hPGCLCs was likely caused by a direct effect of interference with LTR5Hs accessibility and enhancer function (Supplementary Fig. 9A).

Even though significantly fewer hPGCLCs were induced with CRISPRi-LTR5Hs differentiation, no canonical hPGCLC marker was repressed in these cells (Supplementary Fig. 9A). Therefore, to identify potential new LTR5Hs TEENhancer-regulated genes in hPGCLCs, we further analyzed the DEGs up-regulated with hPGCLC differentiation from hESCs and the DEGs down-regulated in CRISPRi-LTR5Hs hPGCLCs compared with CRISPRi-empty hPGCLCs (Supplementary Fig. 9B and Supplementary Data 14). We identified significant overlap (95/124, 76.6% for CRISPRi-LTR5Hs down-regulated DEGs) between down-regulated DEGs in CRISPRi-LTR5Hs hPGCLCs and hPGCLC-specific up-regulated DEGs (Supplementary Fig. 9B and Supplementary Data 14). We thus reasoned that those 95 genes were likely to be the direct targets of LTR5Hs TEENhancers and we predicted that these genes might have a role in hPGCLC biology. For instance, *PRODH, ST6GAL1*, and *STOM* were candidate genes specifically expressed in hPGCLCs, repressed in CRISPRi-LTR5Hs hPGCLCs, and showed hPGCLC specificity relative to somatic cells at single-cell resolution. In addition, these genes were potentially regulated by LTR5Hs TEENhancers (Fig. 4D and Supplementary Fig. 9C, D).

## Discussion

Despite having been initially coined “controlling elements” by Barbra McClintock, TEs were long discarded as parasitic genetic elements. Within the last decade it has become apparent that TEs contribute profoundly to the regulatory landscape of the human genome. Although many TEs are epigenetically silenced by defensive mechanisms, some TE sequences are domesticated by the host during evolution and are therefore kept under selective pressure^24^. *Hominidae*-specific TEs are relatively recent and do not exist in new world-monkeys or even old-world monkeys such as the rhesus or cynomologous macaque. These relatively new *Hominidae* TEs have evolved species-specific functions which are unique to apes, and in some cases are unique to humans.

Here we have shown that one of these *Hominidae*-specific elements, LTR5Hs, is detected at the RNA level both in vitro in hPGCLCs and in vivo in hPGCs using single-cell RNA-seq data from a CS7 human embryo. Using our in vitro model, we likewise show that LTR5Hs elements acquire an open chromatin state, are hypomethylated and bound by key PGC TFs including NANOG, TFAP2C, SOX17, and SOX15 after hPGCLC induction. Further supporting the role of LTR5Hs as enhancers necessary for germ cell specification, we found that LTR5Hs elements are decorated with H3K27ac in hPGCLCs, and that silencing of LTR5Hs using CRISPRi reduces the efficiency of hPGCLC induction, in part due to loss of LTR5Hs enhancer function. Together these results show that *Hominidae*-specific LTR5Hs could serve as TEENhancers necessary for hPGC specification (Fig. 5H), and therefore could be considered essential to successful human reproduction.

Both HERVK-associated and solo-LTR5Hs integrants function as TEENhancers necessary for the maintenance of naïve pluripotency^29,30^. In the naïve state, LTR5Hs copies are hypomethylated, marked with H3K27ac and are synergistically bound by OCT4, p300, and key KLF-family members, most notably KLF4 and KLF17^29,30^. While OCT4 is expressed in both primed and naïve pluripotency, KLF17 and KLF4 are naïve-specific TFs, suggesting that binding by KLF4-KLF17 is necessary for LTR5Hs TEENhancer function in naïve hESCs. Further supporting evidence for this conclusion is the observation that over expression of KLF4-KLF17 in the primed state of pluripotency is sufficient to open the LTR5Hs TEENhancers and regulate neighboring gene expression^30^. Our recent study suggests that KLF17 is not expressed in hPGCLCs, whereas KLF4 is up-regulated upon hPGCLC induction but is not functionally required for the induction or proliferation of hPGCLCs^52^. Thus, we propose that unlike naïve pluripotent stem cells where KLF4 and KLF17 bind to TEENhancers, the LTR5Hs TEENhancers in hPGCLCs utilize SOX17, SOX15, TFAP2C, NANOG, and ETV5. These data collectively argue that while LTR5Hs copies function as TEENhancers in both naïve hESCs and hPGCLCs, the TF networks that reinforce LTR5Hs TEENhancer function in each cell state are distinct.

Despite lack of KLF4 activity and KLF17 expression in germ cell specification, hPGCLCs in vitro and hPGCs in vivo exhibit a naïve-like pluripotent molecular program that has similarities to the naïve state in pre-implantation human embryos. This includes two active X chromosomes in females, genome-wide DNA demethylation and expression of naïve pluripotent TFs including KLF4, TFCP2L1, and TFAP2C^9,16,52–54^. Similar to KLF4-KLF17, TFAP2C also regulates transcription and the identity of naïve pluripotent stem cells by opening naïve-specific enhancers to regulate neighboring gene expression^17,30^. Our results imply that the commissioning of LTR5Hs TEENhancers during hPGCLC induction is driven by the marking of these sites in primed hESCs by a basic network of TFAP2C and NANOG, which is then reinforced with the recruitment of SOX17 and SOX15 during hPGCLC induction. Further supporting this interpretation, time-resolved ATAC-seq during hPGCLC induction from Wang *et al*. shows Sox15 and Oct4:Sox17 motifs become preferentially open during the second day of the four-day hPGCLC differentiation protocol^7^, roughly concomitant with enrichment of naïve-state gene profiles by hPGCLCs^2^. Thus, proper LTR5Hs TEENhancer activity may be necessary for acquisition of a naïve-like transcriptome during hPGCLC induction.

Interestingly, we also observed strong enrichment of ets-ebox binding motifs in hPGCLC-ORs (Supplementary Fig. 7A), which are bound by ETS-family TFs, including ETV4 and ETV5. Recently, it has been proposed that ETV5 is necessary for hPGCLC maintenance, functioning downstream of SOX15. In the absence of SOX15, ETV5 expression is reduced and, reciprocally, efficiency of hPGCLC induction is reduced in the absence of ETV5^7^. These data lead us to hypothesize that ETV5 may also bind LTR5Hs elements during or after hPGCLC induction, possibly following SOX15-mediated commissioning of LTR5Hs enhancers.

We have found that TFAP2C and NANOG are bound to LTR5Hs in undifferentiated hESCs. Our data established a model whereby TFAP2C and NANOG license LTR5Hs in hESCs, and following entry into hPGCLC differentiation, SOX17 and SOX15 cooperate with TFAP2C and NANOG to recruit chromatin remodeling complexes to open chromatin and promote DNA demethylation at LTR5Hs, thus enabling their activity as enhancers. Our results also suggest that localized DNA demethylation over LTR5Hs precedes the global DNA demethylation in the germline, which is a hallmark of hPGC development in the embryo^9,53,54^, further implicating proper commissioning of LTR5Hs enhancer elements as an essential step in, and not a consequence of, hPGCLC induction.

Curiously, despite strong sequence conservation between SVAD elements and the 3′ end of LTR5Hs elements (Fig. 3D), we observe distinct differences in the epigenetic state of these subfamilies after hPGCLC induction. SVAD integrants show less extensive DNA demethylation and accessibility in hPGCLCs with SVAD transcripts being expressed at low levels in hPGCLCs. It has become increasingly appreciated that enhancer elements are often produced by bi-directional, unspliced and often non-polyadenylated RNA Polymerase II-transcribed RNAs, termed enhancer (e) RNAs^55–57^, and that eRNA transcription levels are often positively correlated with the transcriptional levels of nearly genes^55^. Although the function of eRNAs remains enigmatic, production of eRNA has become a hallmark of strong enhancer function. Still, non-transcribed enhancers may act as weak enhancers^57^. Given that SVAD is modestly demethylated and has weak enhancement of chromatin accessibility, it remains possible that SVAD may have some weak enhancer activity in hPGCLCs. In contrast, robust LTR5Hs transcript detection, dramatic DNA demethylation, and chromatin accessibility suggest that LTR5Hs elements act as strong enhancers in hPGCLCs.

While hPGCs acquire a naïve-like transcriptome, they do not fully exit primed state, and demonstrate characteristics of both states^2^. While LTR5Hs/HERVK expression has been linked to a naïve state, enrichment of LTR7/HERVH expression has likewise been associated with the primed pluripotent state^58^, although some LTR7 elements show hallmarks of enhancer function in naïve hESCs^30^. Interestingly, while we detected an up-regulation of LTR5Hs expression, we did not observe any changes in LTR7 expression with hPGCLC induction. Despite no change to LTR7 expression, we did observe a modest decrease in NANOG binding and a decrease in H3K27ac enrichment at certain LTR7 copies upon hPGCLC induction. Thus, while LTR7 expression remains unchanged between primed state hESCs and hPGCLCs, LTR7 enhancer function seems to be decommissioned during hPGCLC induction. This suggests that, in some contexts, LTR7 enhancer function might be uncoupled from RNA production at LTR7 loci.

In addition to gene regulation at enhancers and promoters, human ERVs are also known to regulate 3-D chromatin architecture in pluripotent stem cells. Specifically, HERVH is highly expressed in primed pluripotent stem cells, and is involved in maintaining 3-D chromatin structure^59^. Given that HERVH is down-regulated and LTR5Hs sequences are up-regulated during hPGCLC induction, it is possible that LTR5Hs may also be required for the assembly of genome 3-D architecture in hPGCLCs, and therefore the failure to fully repress HERVH in the CRISPRi-LTR5Hs hPGCLCs.

Finally, here we have identified three potential LTR5Hs-regulated genes which may be important to hPGC biology based on their selective expression in hPGCLCs and their down-regulation following CRISPRi-LTR5Hs treatment. Of particular interest is *ST6GAL1*, a sialyltransferase^60^ that produces CD75, a cell-surface glycoprotein that serves as a marker of naïve hESCs^49^. *ST6GAL1* is likewise regulated by LTR5Hs in naïve hESCs^30^, offering further support to our hypothesis that LTR5Hs TEENhancers act to reinforce elements of the naïve transcriptome during hPGCLC/hPGC maintenance. While the role of both *ST6GAL1* and CD75 remains enigmatic, knockdown of *ST6GAL1* during reprogramming of human dermal fibroblast (HDF) impedes reprogramming and causes a delay in the expression of *NANOG*, *OCT4,* and *SOX2* RNA^61^. Conversely, knockdown of *ST6GAL1* in primed hESC had a modest effect on the transcriptome, causing an up-regulation of genes associated with organogenesis^61^. Recent work by Liu et al.^62^. has produced a high-resolution roadmap of the transcriptome during HDF reprogramming, which uncovered an intermediate state immediately prior to a lineage bifurcation between primed and naïve transcriptome acquisition. It is tempting to speculate that *ST6GAL1* may be necessary to efficiently pass through this intermediate state and that during hPGC specification or hPGCLC induction *ST6GAL1* has a similar role as latent pluripotency is re-established following specification or induction, respectively.

Modern and archaic humans began to diverge ~500,000 years ago with modern humans becoming the dominant surviving human species ~50,000 years ago^63,64^. Extinction occurs when reproduction fails. Considering the contributions of TEs to the renewal of the genetic pool during evolution, one hypothesis could be that human-specific TEs, like LTR5Hs became beneficial to germ cell specification and consequently improved human reproductive fitness. As we have identified multiple TF networks that converge on LTR5Hs, it is also possible that other factors not profiled in this work contribute to the specification and reinforcement of PGC fate. Likewise, advances in recent techniques to model the early embryo could provide additional platforms to dissect the networks which delineate the naïve state networks in the pre-implantation embryo.

## Methods

### Ethics statement

The UCLA2 and UCLA1 hESC lines were derived at UCLA by the UCLA Pluripotent Stem Cell Core Facility following Institutional Review Board (IRB) and UCLA Embryonic Stem Cell Research Oversight (ESCRO) Committee Approvals. Informed consent was obtained after the embryo donors contacted the UCLA Broad Stem Cell Research Center to inquire about donating surplus embryos following in vitro fertilization. Embryo donors were not paid and were able to freely withdraw consent to use the embryos for stem cell research up to the point of hESC derivation when the embryo is destroyed. Informed consent was obtained from all embryo donors prior to sending frozen donated embryos to UCLA. Once derived, the hESC lines were authenticated using Affymetrix Genome-wide Human SNP Array 6.0 to detect Single Nucleotide Polymorphisms and Copy Number Variant (SNP/CNV) prior to distribution. The UCLA1 and UCLA2 hESC lines are provided to researchers de-identified, with all links and identifiers broken prior to distribution. All de-identified hESC lines used in this study are registered with the National Institute of Health Human Embryonic Stem Cell Registry and are available for research use with NIH funds. Mycoplasma test (Lonza, LT07-418) was performed every month. All experiments using the de-identified hESC lines were approved by the UCLA Embryonic Stem Cell Research Oversight Committee.

### Cell culture

UCLA2 and UCLA1 hESC are cultured as previously described^16^, briefly the hESCs are cultured in hESC media, which was composed of 20% knockout serum replacement (KSR) (Life Technologies, A3181502), 1x MEM Non-Essential Amino Acids (NEAA) (Fisher Scientific, 25-025-CI), 1x Penicillin/Streptomycin/Glutamine (Thermo Fisher, 10378016), 55 µM 2- Mercaptoethanol (Life Technologies, 21985-023), 10 ng/mL recombinant human FGF basic (Proteintech, HZ1285), and 50 ng/mL primocin (InvivoGen, ant-pm-2) in DMEM/F12 media (GIBCO, 11330-032). The primed hESCs were split by 1 mg/ml Collagenase type IV (GIBCO, 17104-019) and maintained routinely on mitomycin C (MMC)-inactivated mouse embryonic fibroblasts (MEFs). The hESCs were split every 7 days using Collagenase type IV (GIBCO, 17104-019). HEK293 cells were acquired from ATCC (Cat# CRL-3216). No lines used in this study belong to the International Cell Line Authentication Committee register of misidentified cell lines.

### hPGCLC differentiation

Using the UCLA2 hESC line, the differentiation of hPGCLCs in vitro was performed as previously described^4,5^. Specifically, 0.05% trypsin-EDTA (Thermo Fisher Scientific, 25300120) was used to digest confluent hESCs cultured on mitomycin C inactivated mouse embryonic fibroblasts (MEFs) into single cells, followed by plating onto a 12-well-plate that had previously been coated with human plasma fibronectin (Life Technologies, 33016-015) for at least 1 hour (h). Cells were plated at cell density of 200,000 cells/well in 2 mL/well of incipient mesoderm-like cells (iMeLCs) medium, which is composed of 15% knockout serum replacement (KSR, Life Technologies, A3181502), 1x MEM Non-Essential Amino Acids (NEAA) (Fisher Scientific, 25-025-CI), 55 µM 2-Mercaptoethanol (Life Technologies, 21985-023), 1x Penicillin/Streptomycin/Glutamine (Thermo Fisher, 10378016), 1 mM sodium pyruvate (Life Technologies, 11360070), 50 ng/mL Activin A (PeproTech, AF-120-14E), 3 mM CHIR99021 (Reprocell, 04-0004-10), 10 mM ROCKi (Y27632, Stemgent, 04-0012-10), and 50 ng/mL primocin (InvivoGen, ant-pm-2) in Glasgow’s minimal essential medium (GMEM) (Life Technologies, 11710035). After 24 h, iMeLCs were dissociated into single cells by 0.05% trypsin-EDTA (Thermo Fisher Scientific, 25300120), then plated into ultra-low cell attachment U-bottom 96-well plates (Corning, 7007) at a density of 3000 cells/well in 200 mL/well of hPGCLC medium, which is composed of 15% KSR (Life Technologies, A3181502), 1x MEM Non-Essential Amino Acids (NEAA) (Fisher Scientific, 25-025-CI), 55 µM 2- Mercaptoethanol (Life Technologies, 21985-023), 1x Penicillin/Streptomycin/Glutamine (Thermo Fisher, 10378016), 1 mM sodium pyruvate (Life Technologies, 11360070), 10 ng/mL recombinant human leukemia inhibitory factor (Sigma-Aldrich, LIF1010), 200 ng/mL human BMP4 (R&D systems, 314-BP), 50 ng/mL human epidermal growth factor (Fisher Scientific, 236EG200), 10 mM of ROCKi (Y27632, Stemgent, 04-0012-10), and 50 ng/mL primocin in GMEM (Life Technologies, 11710035). Day-4 hPGCLC aggregates were collected for further analysis.

### Flow cytometry and fluorescence-activated cell sorting

hPGCLC aggregates were dissociated with 0.05% Trypsin-EDTA (Thermo Fisher Scientific, 25300120) for 10 minutes (min) at 37 °C. The dissociated cells were then stained with conjugated antibodies, washed with FACS buffer (1% BSA in PBS) and resuspended in FACS buffer with 7-AAD (BD PharMingen, 559925) as viability dye. The single-cell suspension was sorted for further experiments. For SOX17 ChIP-seq, all hPGCLCs were collected and sorted by BD FACSDiva v8.0.2. For NANOG ChIP-seq, 96 aggregates were sampled via FACS, while the remaining aggregates were dissociated in parallel before being fixed and flash frozen (see TF Chromatin Immunoprecipitation). The antibodies used in this study are: BV421 conjugated anti-human/mouse CD49f (ITGA6) (BioLegend; Cat#313624; RRID: AB_2562244; Lot#B274314) at 1/80; APC-conjugated anti-human CD326 (EPCAM) (BioLegend; Cat#324208; RRID: AB_756082; Lot#B284158) at 1/80.

### ChIP-seq protocol

The ChIP-seq was performed as previously described^17^. Isolated hPGCLCs (SOX17) or whole hPGCLC aggregates (NANOG) were fixed using 1% formaldehyde (Thermo Fisher Scientific, Waltham MA) rotating at room temperature for 10 min. Fixation was quenched using 0.14 M Glycine (Sigma Aldrich, St. Louis MO), cells were pelleted by centrifugation at 3000 RPM for 5 min. Resulting cell pellets were flash frozen in liquid nitrogen and stored at −80 °C prior to immunoprecipitation.

Pellets were thawed on ice and resuspended in lysis buffer (10 mM Tris HCl pH 8, 0.25% Triton-X 100, 10 mM EDTA, 0.5 mM EGTA, supplemented with Halt Protease Inhibitor cocktail (Thermo Fisher Scientific, Waltham, MA)) at room temperature, rotating, for 15 min. The resulting lysate was pelleted by 5 min of centrifugation at 4000 RPM. Pellet was resuspended in Nuclei isolation buffer (10 mM Tris-HCl pH 8.0, 200 mM NaCl, 10 mM EDTA, 0.5 mM EGTA supplemented with Halt Protease Inhibitor Cocktail (Thermo Fisher Scientific, Waltham MA)) at 4 °C, rotating for 10 min followed by 5 min of centrifugation at 4000 RPM. The resulting pellet was resuspended in 10 mM Tris HCl pH 8, 10 mM EDTA, 0.5 mM EGTA with protease inhibitors. Samples were sonicated using a Covaris (Woburn, MA) S220 (Intensity of 5, 200 cycles per burst, 5% duty cycle) in 8 cycles of 30 seconds on, 30 seconds off for an effective 4 min of sonication. Insoluble material was removed by centrifugation at 14,000 RPM for 10 min at 4 °C. In all, 10% of resulting soluble supernatant was saved as an input sample. To pre-clear, Protein A beads (30 μL/ sample) were washed in dilution buffer (16.7 mM Tris-HCl pH 8, 0.01% SDS, 1.1% TritonX-100, 1.2 mM EDTA, 167 mM NaCl) for three times. Protein A beads were resuspended in dilution buffer and added to samples so that 30 μL of Protein A Dynabeads were suspended in a volume of dilution buffer equal to the volume of soluble material. Chromatin was pre-cleared by incubation with Protein A Dynabeads (Thermo Fisher, Waltham MA) for 2 h, rotating at 4 °C. Beads were removed and 1.6 μg of anti-SOX17 antibody (Cat#AF1924, R and D Systems) or 1.2 ug of anti-NANOG antibody (Cat#AF1997, R and D Systems) were added and allowed to incubate rotating at 4 °C overnight. Antibodies bound using 60 μL of Protein G Dynabeads by incubation at 4 °C, rotating for 2 h. Antibody-bound beads were washed 2 × 4 min with 50 mM HEPES pH 7.9, 1% TritonX-100, 0.1% Deoxycholate, 1 mM EDTA, 140 mM NaCl at room temperature, followed by 2 washes with 50 mM HEPES pH 7.9, 0.1% SDS, 1% TritonX-100, 1 mM EDTA, 500 mM NaCl. Beads were subsequently washed twice with 10 mM Tris HCl, pH 8, 1 mM EDTA. Chromatin was eluted from beads by heating 65 °C, rotating at 1400 RPM in 50 mM Tris HCl pH 8, 1 mM EDTA, 1% SDS twice. To facilitate crosslinking reversal, eluate was left to incubate at 65 °C overnight. Eluate was treated with 15 μg RNase A at 37 °C for 30 min followed by treatment with 100 μg of Proteinase K at 56 °C. DNA was purified using Qiagen PCR purification kit according to manufacturer’s instructions.

Eluted DNA was used to generate libraries for ChIP-seq using Tecan Genomics Ovation UltraLow V2 DNA-seq (0344NB, Redwood City, CA) according to the manufacturer’s instructions. All ChIP-seq libraries were sequenced using a NovaSeq 6000 (Illumina, San Diego) on an NovaSeq SP lane using paired-end 100 base pair reads.

### CRISPR/dCas9-kRAB assay

Two gRNAs (gRNA55 and gRNA57) that targeted LTR5Hs were designed by Pontis and colleagues^30^. The two gRNAs were cloned into pLV-dCas9-KRAB-T2a-Puro (Addgene 71236), and the plasmid with no LTR5Hs gRNA was used as a control. Using a second-generation lentiviral system we generated dCas9-KRAB-gRNA55, dCas9-KRAB-gRNA57, and dCas9-KRAB-empty virus in HEK293T cells (ATCC, Manassas, Virginia, Cat# CRL-3216). Supernatants that contain lentivirus were then collected and ultracentrifuged. Confluent hESC were trypsinized with 0.05% trypsin at 37 °C for 5 min, then 200k cells were counted and collected to mix with the concentrated lentivirus. After mixture on nutator for two hours at room temperature, cells were transferred onto mitomycin C treated MEFs in hESC media with 10 mM ROCKi. After transduction into hESC, 1ug/mL puromycin was used to screen for positive cells for at least 5 days. Surviving cells were then used to perform downstream assays.

### RNA-sequencing

RNA-seq was performed as previously described^65^. Briefly the hPGCLCs were directly sorted into 350 μL RLT lysis buffer (QIAGEN RNeasy micro kit, 220006-800). Total RNA was then extracted by RNeasy micro kit (Qiagen RNeasy micro kit, 220006-800). Total RNA was reverse transcribed and cDNA was amplified using Ovation RNA-Seq System V2 (Tecan, 7102-32) according to the manufacturer’s instructions. Amplified cDNA was then sheared to ~200 bp length by Covaris S220 Focused ultrasonicator. RNA-seq libraries were constructed by using Ovation Rapid Library Systems (Tecan, 0319-32) and quantified by a KAPA library quantification kit (Kapa Biosystems, kk4824). Libraries were then subjected to pair-end sequencing on Illumina NovaSeq 6000 sequencer.

### Statistics and reproducibility

No statistical method was used to predetermine sample size and no data were excluded from the analyses. For CRISPRi experiments, hESCs from within a given cell line were pooled and randomly allocated to either CRISPRi-virus or control-virus conditions. ChIP-seq experiments were not randomized. Authors were not blinded to allocation during experiments and outcome assessment. All statistics were calculated using GraphPad Prism v9.2.0 (283) or R^66^ v3.5.1 unless otherwise mentioned in the figure legend. The Statistical test methods used were provided in Source Data file.

### Bioinformatics analysis

#### Reference genome

Human reference genome GRCh38.97 from Ensembl^67^ was used for STAR^36^ v2.7.0e and BSMAP^68^ v2.74 alignment, while human reference genome hg38 from UCSC^69^ was used for SQuIRE^32^ v0.9.9.92 for alignment. TE annotation file from repeatmasker (http://repeatmasker.org/) GRCh38 and gene annotation file from Ensembl^67^ GRCh38.97 was utilized for all genomics analysis.

#### TE quantification methods comparison

Four methods for TE quantification were applied to call DETEs in hPGCLCs compared with hESCs, to identify TE subfamilies specific to hPGCLCs more precisely. RNA-seq data of hESC to hPGCLC differentiation was from previous publication^16^ GSE93126 (Supplementary Data 1).

Quality control for raw RNA-seq sequences was performed by FastQC^70^ v0.11.8. Then the raw reads were aligned by STAR^36^ v2.7.0e or SQuIRE^32^ v0.9.9.92. For STAR alignment, maximal 1000 multiple mapped reads were allowed, and the best hit was kept (--outFilterMultimapNmax 1000 --outSAMmultNmax 1). SQuIRE Map function with default parameters was applied for alignment. The output bam format files were sorted and indexed by SAMtools^71^ v1.9 for downstream analysis. Bigwig tracks were generated using deeptools^72^ v3.4.3 by normalizing to RPKM (Reads Per Kilobase per Million mapped reads) using bin size of 10 bp.

Read quantification for individual TE copies were calculated using featureCounts^35^ v2.0.0, SQuIRE^32^ v0.9.9.92, Telescope^34^ v2.0.0, or TEtranscripts^33^ v2.2.1. FeatureCounts, Telescope and TEtranscripts used the sorted bam file from STAR, while SQuIRE used its own sorted bam file. Multiple mapped reads were included for TE quantification (featureCounts -M, TEtranscripts --mode multi, SQuIRE, and Telescope using default parameters). Differential expressed TEs (DETEs) were processed using R package DESeq2^37^ v1.26.0 for the count matrices from TE quantification. Only TE with RPKM mean in either control or treatment group >1 were kept for further analysis. DETEs were obtained with at least 4-fold change and FDR < 0.05.

#### RNA-seq analysis

Other than methods used for the TE quantification, “STAR + featureCounts + DESeq2” method was applied for both TE and gene quantification and DETE/DEG calling in the article. Besides GSE93126 RNA-seq data^16^ of hESC to hPGCLC differentiation (Supplementary Data 1), other RNA-seq datasets used in this article including RNA-seq of CRISPRi in hPGCLCs generated from this paper, RNA-seq data of hESC multilineage differentiation from previous publication^41^ GSE16256 (Supplementary Data 1), and RNA-seq data of CRISPRi in naïve hESCs from previous publication^30^ GSE117395 (Supplementary Data 1).

For RNA-seq data quality control, alignment and track generation, FastQC^70^ v0.11.8, STAR^36^ v2.7.0e and deeptools^72^ v3.4.3 were applied as described in “TE quantification methods comparison” section.

Both gene and TE were quantified using FeatureCounts^35^ v2.0.0, with “-M” option allowing the quantification for multiple mapped reads. For DETEs and DEGs calling by DESeq2^37^ v1.26.0, only TE or gene with RPKM mean in either control or treatment group >1 were kept for further analysis. DETEs were obtained with at least 4-fold change and FDR < 0.05 while DEGs were obtained with at least 1.5-fold change and FDR < 0.05.

To visualize the top 200 TE subfamilies that are most dynamically expressed in hESCs, iMeLCs, and hPGCLCs, top 200 TE subfamilies with the largest variance for the normalized counts across the three cell types were kept. *Z*-score of the RPM (Reads Per Million mapped reads) for each TE subfamily was used for data visualization.

To analyze the expression level in hESCs and hPGCLCs over HERV associated LTR or solo LTRs, we classified solo LTR5Hs and solo LTR7 as following. The distance between LTR5Hs (or LTR7) individual copy to the nearest HERVK (or HERVH) was first calculated using bedtools^73^ v2.29.2 closest function. The distance distribution was then summarized in R^66^ v3.5.1. LTR5Hs within 100 bp distance to nearest HERVK were classified as HERVK-LTR5Hs, while others were defined as solo LTR5Hs. LTR7 within 10 bp distance to nearest HERVH were classified as HERVH-LTR7, while others were defined as solo LTR7.

#### scRNA-seq analysis

Two biological replicates for the scRNA-seq data of hESC to hPGCLC differentiation (UCLA2 line) was downloaded from previous publication^2^ GSE140021 (Supplementary Data 1). The reads were quantified by 10x Genomics Cell Ranger^74^ v.3.1.0 to both gene and TE reference genome with default parameters. The generated cell-by-gene/TE unique molecular identifier (UMI) count matrix was analyzed in Seurat^75^ R package v3.2.2. Due to limited coverage in scRNA-seq data, we aggregated reads from individual TE copies to TE subfamilies for downstream analysis.

Cells expressing 1000–7000 gene features and <20% mitochondrial genes were kept. The UMI counts were then normalized and log-transformed followed with identifying top 2000 variable features and scaling for both gene and TE UMI count matrix with default parameter. For batch correction between two replicates, we used Seurat’s IntegrateData function with default parameter, which were used further for clustering and UMAP visualization. The scaled integrated data with variable genes was used to perform principal component analysis (PCA). UMAPs were calculated by RunUMAP function using top 50 principal components and resolution 1.

Raw data for scRNA-seq of two Carnegie Stage 7 human gastrula embryos was kindly shared by the authors from previous publication^40^ (Supplementary Data 1). Single-cell RNA-seq data of seven PGCs as well as other randomly selected cells from annotated cell types (epiblast, primitive streak, emergent mesoderm and advanced mesoderm, annotated by Tyser et al.), were re-analyzed for gene and TE expression same as “STAR + FeatureCounts” RNA-seq analysis method. In brief, FastQC^70^ v0.11.8 was used for quality control, STAR^36^ v2.7.0e was used for alignment, both gene and TE were quantified by FeatureCounts^35^ v2.0.0, with “-M” allowing multiple mapping for TEs.

#### ATAC-seq analysis

Raw ATAC-seq data from previous publication^16^ GSE120648 (Supplementary Data 1) were downloaded followed by quality control with FastQC^70^ v0.11.8. Then raw reads were aligned by STAR^36^ v2.7.0e allowing maximal 1000 multiple mapped reads with no more than three mismatches and the best hit was kept (--outFilterMultimapNmax 1000 --outFilterMismatchNmax 3 –outSAMmultNmax 1). Splice junction was neglected by building STAR index without general feature format file and not allowing intron length (--alignIntronMax 1). PCR duplicates were removed using SAMtools^71^ v1.9 rmdup function. SAMtools^71^ v1.9 merge function was used to merge aligned reads in bam format for replicates in each cell type for downstream analysis to increase coverage.

ATAC-seq peaks were defined using the MACS2^76^ v2.2.7.1 callpeaks function. Here we only kept peaks with a fold change enrichment >4 from the MACS2 output. In order to identify hPGCLC- or hESC-ORs, we used bedtools^73^ v2.29.2 multiinter function with Ryan Layers’s clustering, and the regions <100 bp were discarded. Bigwig tracks were generated using deeptools^72^ v3.4.3 by normalizing to RPKM using binsize of 10 bp. ATAC-seq signal over hPGCLC- or hESC-ORs or TE regions were visualized using deepTools^72^ v3.4.3.

To quantify ATAC-seq signals over LTR5Hs as well as random shuffled regions, we first generated 100,000 random shuffled TE and genomic regions. A hundred thousand (*n* = 100,000) TE random regions were randomly selected 100,000 TE individual copies from all TE copies in human reference genome. A hundred thousand (*n* = 100,000) genome random regions were randomly shuffled genomic regions with the same length as 100,000 TE individual copy regions generated by bedtools^73^ v2.29.2 shuffle function. The ATAC-seq read counts over LTR5Hs, 100,000 random shuffled TE and genomic regions were calculated with bedtools^73^ v2.29.2 multicov function. Then, read counts were normalized to total reads aligned in each sample using RPM and visualized in R^66^ v3.5.1.

#### TE enrichment analysis over hPGCLC- or hESC-ORs

TE annotation for hPGCLC- or hESC-ORs was conducted by Homer^77^ v4.7 annotatePeaks.pl function using GRCh38 TE annotation file.

To generated randomly shuffled regions with comparable genomic distribution to TEs, random shuffled regions for ATAC-seq hPGCLC- or hESC-ORs were adjusted by the relative proportion of genomic regions (promoter, exon, intron, TTS, 10 kb gene proximal region, 10–100 kb distal region or >100 kb intergenic region), according to Chuong et al.^43^. To be specific, the midpoints of hPGCLC/hESC-ORs were annotated to genomic regions (promoter, exon, intron, TTS, intergenic region) by Homer^77^ v4.7 annotatePeaks.pl function. Then intergenic region was further divided into 10 kb gene proximal region, 10–100 kb distal region or >100 kb intergenic region by their distance to the nearest gene. Then, the entire human genome was divided into promoter, exon, intron, TTS, intergenic region (10 kb gene proximal region, 10–100 kb distal region or >100 kb intergenic region). The annotated midpoints of hPGCLC/hESC-ORs in each kind of genomic region were shuffled 10,000 times within the corresponding genomic region with bedtools^73^ v2.29.2 shuffle function (-seed 1 to 10,000) by keeping the shuffled regions on the same chromosome (-chrom). Then, the shuffled regions with same seed number were merged to create shuffled hPGCLC/hESC-ORs maintaining the same genomic distribution as the original hPGCLC/hESC-ORs.

The expected TE occurrence was calculated by the average number of TE copies which were intersected with the 10,000 combined random shuffled hPGCLC/hESC-ORs. If the expected TE copy occurrence for certain TE subfamily was smaller than 1, it was rounded to 1. The observed TE occurrence was counted based on the TE annotation for hPGCLC/hESC-ORs. The value of Log_2_ transformed “observed TE occurrence/expected TE occurrence” was used as enrichment score, with one-sided exact binomial test for statistical test.

#### Transcription factor motif enrichment analysis

Motif file for over 400 transcriptional factors were collected from Homer^77^ v4.7 and the position of each motif in GRCh38 genome were calculated using Homer scanMotifGenome.pl function. Next, hPGCLC-ORs overlapped with LTR5Hs were identified by bedtools^73^ v2.29.2 intersect function. As control, those LTR5Hs overlapped hPGCLC-ORs were randomly distributed using bedtools^73^ v2.29.2 shuffle function while keeping on the same chromosome (-chrom).

To analyze the enrichment of TF motifs over chromatin opened LTR5Hs, the frequency of occurrences for TF motifs in hPGCLC-ORs overlapped LTR5Hs and shuffled control were processed using bedtools^73^ v2.29.2 intersect function. Top 50 TF motifs with highest enrichment ratios were plotted.

#### WGBS analysis

Raw WGBS data were downloaded from previous publication^44^ GSE139115 (Supplementary Data 1). Reads were aligned with BSMAP^68^ v.2.74 by mapping reads to all four strands (-n 1), allowing maximum one equal best hits and less than two mismatches per read (-w 1, -v 2). Aligned reads in bam format for biological replicates of hESC and hPGCLCs were merged to increase the coverage using SAMtools^71^ v1.9 merge function. Methratio.py script built in BSMAP were used to calculate cytosine counts only keeping unique mappings (-u), non-duplicated reads (-r), and reporting loci with zero methylation ratios (-z). Methylation level at CG sites was then calculated by #C/(#C + #T).

To visualize the CG methylation level and cytosine coverage over LTR5Hs and SVAD, only CG sites with ≥3 covered reads were retained. Wiggle tracks were generated with customized perl script and converted to bigwig with wigToBigWig^78^ v4 followed by data visualization with deeptools^72^ v3.4.3.

To analyze CG methylation level over LTR5Hs and its related TE clades obtained from the Dfam^45^ database that shared the most sequence similarity, #C and #C + #T count over each individual TE copy were extracted with customized python script and plotted in R.

DMR were defined using R package DMRcaller^79^ v1.14.2 over GRCh38 whole genome using 200 bp as DMR bin size. Only bins with at least four CG sites and each CG sites should be covered by at least three reads were kept for further analysis. Minimal CG methylation difference of 0.2 and FDR less than 0.05 were applied to define DMRs. Bins defined as DMR and within 100 bp gap were merged.

#### ChIP-seq analysis

NANOG and SOX17 ChIP-seq data in hESCs and hPGCLCs were generated in this paper. TFAP2C ChIP-seq in hESCs and hPGCLCs, H3K27ac ChIP-seq in hPGCLCs were from previous publication^2^ GSE140021 (Supplementary Data 1). H3K27ac ChIP-seq in hESCs was from previous publication^48^ GSE69646 (Supplementary Data 1). SOX15 CUT&Tag-seq was from previous publication^7^ GSE143345 (Supplementary Data 1).

For all ChIP-seq data, quality control was performed by FastQC^70^ v0.11.8. Then reads were aligned by STAR^36^ v2.7.0e allowing maximal 1000 multiple mapped reads with no more than three mismatches and the best hit was kept (--outFilterMultimapNmax 1000 --outFilterMismatchNmax 3 –outSAMmultNmax 1). Splice junction is neglected by building STAR index without general feature format file and not allowing intron length (--alignIntronMax 1). PCR duplicates were removed using SAMtools^71^ v1.9 rmdup function.

Representative replicate for each condition was used for downstream analysis. ChIP-seq peaks were defined using the MACS2^76^ v2.2.7.1 callpeaks function by setting ChIP file as treatment and input file as control. Bigwig tracks were generated using deeptools^72^ v3.4.3 by normalizing to RPKM using binsize of 10 bp. ChIP-seq signal over TE regions were visualized using deeptools^72^ v3.4.3. Motif annotation over ChIP-seq peak summits used Homer^77^ v4.7 findMotifsGenome.pl function with fragment size 200 and masking repeats (-size 200 -mask). TF-bound LTR5Hs/LTR7 copies were identified by bedtools^73^ v2.29.2 intersect function.

#### CRISPRi gRNA target sites prediction

To search the predicted sites of LTR5Hs targeting gRNA, we used the Homer^77^ v4.7 to generate motif file for gRNA plus PAM NGG sequence (CTCCCTAATCTCAAGTACCCNGG, TGTTTCAGAGAGCACGGGGTNGG) using seq2profile.pl and searched the targeting sites using scanMotifGenomeWide.pl with <3 mismatches. The target sites were annotated using gene and TE annotation and then categorized into either promoter, exonic, TE, intronic, or intergenic sites. If one target site was annotated with multiple categories, only one category would be retained with priority order of promoter, exon, TE, intron, and intergenic sites.

#### RAD analysis

For RAD analysis, we used the website application from Guo et al.^51^. For RAD analysis of LTR5Hs associated DEGs, up- and down-regulated DEGs in CRISPRi-LTR5Hs were input as DEGs lists; LTR5Hs bed file or randomly shuffled LTR5Hs bed file by bedtools^73^ v2.29.2 shuffle function were input as Genomic Regions of Interest (gROI) file. For RAD analysis of CRISPRi gRNAs predicted sites associated DEGs, up- and down-regulated DEGs in CRISPRi-LTR5Hs were input as DEGs lists; bed file of CRISPRi gRNAs predicted sites was input as gROI file. For submit options, “GRCh38” was chose for reference genome, “1000, 800, 600, 400, 200, and 0 kb” was input as customized peak extend distance correspondingly, “hypergeometric test” was choosing for statistical test.

### Reporting summary

Further information on research design is available in the Nature Research Reporting Summary linked to this article.

## Supplementary information

Supplementary information

Reporting Summary

Description of Additional Supplementary Files

Supplementary Data 1

Supplementary Data 2

Supplementary Data 3

Supplementary Data 4

Supplementary Data 5

Supplementary Data 6

Supplementary Data 7

Supplementary Data 8

Supplementary Data 9

Supplementary Data 10

Supplementary Data 11

Supplementary Data 12

Supplementary Data 13

Supplementary Data 14

## Data Availability

All high-throughput sequencing data generated are accessible at NCBI’s Gene Expression Omnibus (GEO) via GEO Series accession number GSE182218. Source data are provided with this paper.

## Source data

Source Data

## Code availability

Customized code/scripts used in this study are available from the corresponding author upon request.
