# Supplementary material for: Human reproduction is regulated by retrotransposons derived from ancient Hominidae-specific viral infections
Source: Nat Commun. 2022 Jan 24;13:463. doi: 10.1038/s41467-022-28105-1 (PMC8786967; doi:10.1038/s41467-022-28105-1)
Supplement: Supplementary file 1 — Supplementary information [file 41467_2022_28105_MOESM1_ESM.pdf]

Supplementary Information

Human Reproduction is Regulated by Retrotransposons derived from Ancient Hominidae-Specific Viral Infections

Xinyu Xiang[1,10], Yu Tao[2,10], Jonathan DiRusso[2,3], Fei-Man Hsu[2], Jinchun Zhang[1], Ziwei Xue[1], Julien Pontis[4], Didier Trono[4], Wanlu Liu[1,5,6,7,†], Amander T. Clark[2,3,8,9,†]

1. Zhejiang University-University of Edinburgh Institute (ZJU-UoE Institute), Zhejiang University School of Medicine, International Campus, Zhejiang University, 718 East Haizhou Rd., Haining, 314400, China
2. Department of Molecular Cell and Developmental Biology, University of California, Los Angeles, Los Angeles, CA 90095, USA
3. Molecular Biology Institute, University of California, Los Angeles, Los Angeles, CA 90095, USA
4. School of Life Sciences, Ecole Polytechnique Fe´de´rale de Lausanne (EPFL), 1015 Lausanne, Switzerland
5. Department of Orthopedic Surgery of the Second Affiliated Hospital of Zhejiang University School of Medicine, Zhejiang University, Hangzhou, 310029, China
6. Dr. Li Dak Sum & Yip Yio Chin Center for Stem Cell and Regenerative Medicine, Zhejiang University, Hangzhou, Zhejiang 310058, China
7. Alibaba-Zhejiang University Joint Research Center of Future Digital Healthcare, Zhejiang University, Hangzhou, Zhejiang 310058, China
8. Eli and Edythe Broad Center of Regenerative Medicine and Stem Cell Research, University of California, Los Angeles, Los Angeles, CA 90095, USA
9. Jonsson Comprehensive Cancer Center, University of California, Los Angeles, Los Angeles, CA 90095, USA
10. These authors contributed equally to this work.

† Corresponding author. Email: wanluliu@intl.zju.edu.cn (W.L.); clarka@ucla.edu (A.C.)

A

Reference genome	Alignment: Software	Alignment: Parameters	TE count: Software	TE count: Parameters	DETE calling: Software	DETE calling: Parameters	Citation
GRCh38.97	STAR v.2.7.0e	--outFilterMultimapNmax 1000 --outSAMmultNmax 1	featureCounts v.2.0.0	-M	DESeq2 v.1.26.0	$\lvert Log_2FC \rvert \geq 2$ FDR ≤ 0.05 RPKM mean* ≥ 1	*Dobin et al., 2013* *Liao et al., 2014* *Love et al., 2014*
GRCh38.97	SQuIRE v.0.9.9.92	-r 51 -b hg38	SQuIRE v.0.9.9.92	-r 51 -b hg38	DESeq2 v.1.26.0	$\lvert Log_2FC \rvert \geq 2$ FDR ≤ 0.05 RPKM mean* ≥ 1	*Yang et al., 2019* *Love et al., 2014*
GRCh38.97	STAR v.2.7.0e	--outFilterMultimapNmax 1000 --outSAMmultNmax 1	Telescope v.2.0.0	--attribute transcript_id	DESeq2 v.1.26.0	$\lvert Log_2FC \rvert \geq 2$ FDR ≤ 0.05 RPKM mean* ≥ 1	*Dobin et al., 2013* *Bendall et al., 2019* *Love et al., 2014*
GRCh38.97	STAR v.2.7.0e	--outFilterMultimapNmax 1000 --outSAMmultNmax 1	TEtranscripts v.2.2.1	--mode multi	DESeq2 v.1.26.0	$\lvert Log_2FC \rvert \geq 2$ FDR ≤ 0.05 RPKM mean* ≥ 1	*Dobin et al., 2013* *Jin et al., 2015* *Love et al., 2014*

*RPKM mean: the RPKM mean of either control group or treatment group is greater than 1.

B

C

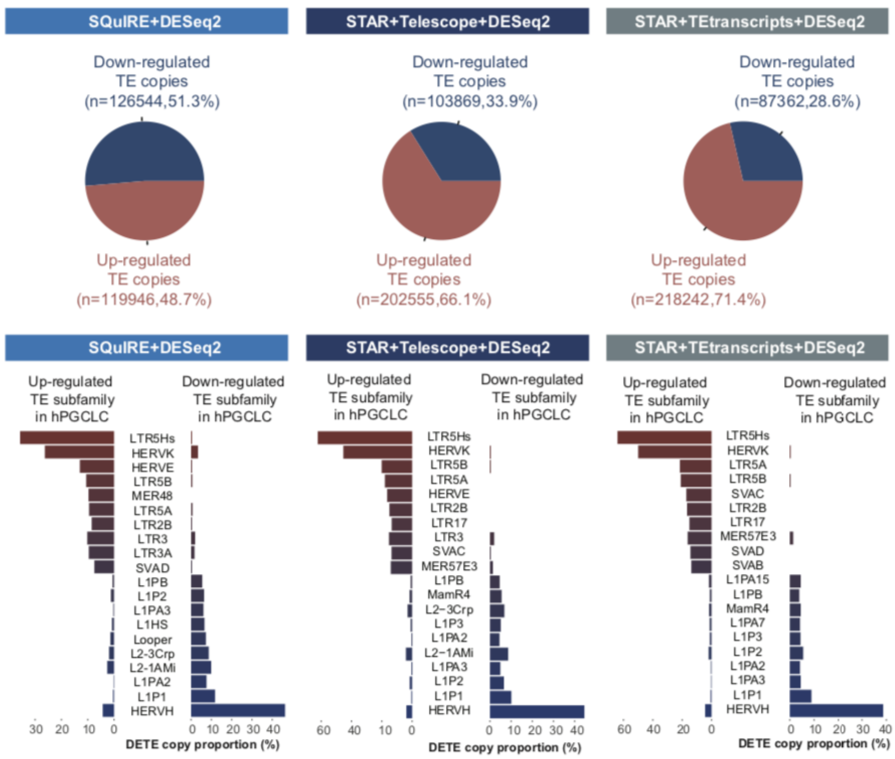

Figure S1. Comparison of different methods for TE quantification in RNA-seq data.

A. The workflow and parameters used in the four major TE quantification methods we tested. **B.** Pie chart showing the proportion of up- or down-regulated DETE copies in hPGCLCs compared to hESCs processed by different methods (left panel: SQuIRE+DESeq2; middle panel: STAR+Telescope+DESeq2; right panel: STAR+TEtranscripts+DESeq2) with at least 4-fold changes and FDR less than 0.05 as cut-off. **C.** Top ten up- or down-regulated TE subfamilies in hPGCLCs processed by different methods (left panel: SQuIRE+DESeq2; middle panel: STAR+Telescope+DESeq2; right panel: STAR+TEtranscripts+DESeq2). X axis shows proportion of DETE relative to the total copy number for each TE subfamily. Only TE subfamilies with at least 80 copies and 8 DETE copies are kept for this

analysis. Source data underlying Supplementary Fig. 1C is provided as a Source Data file.

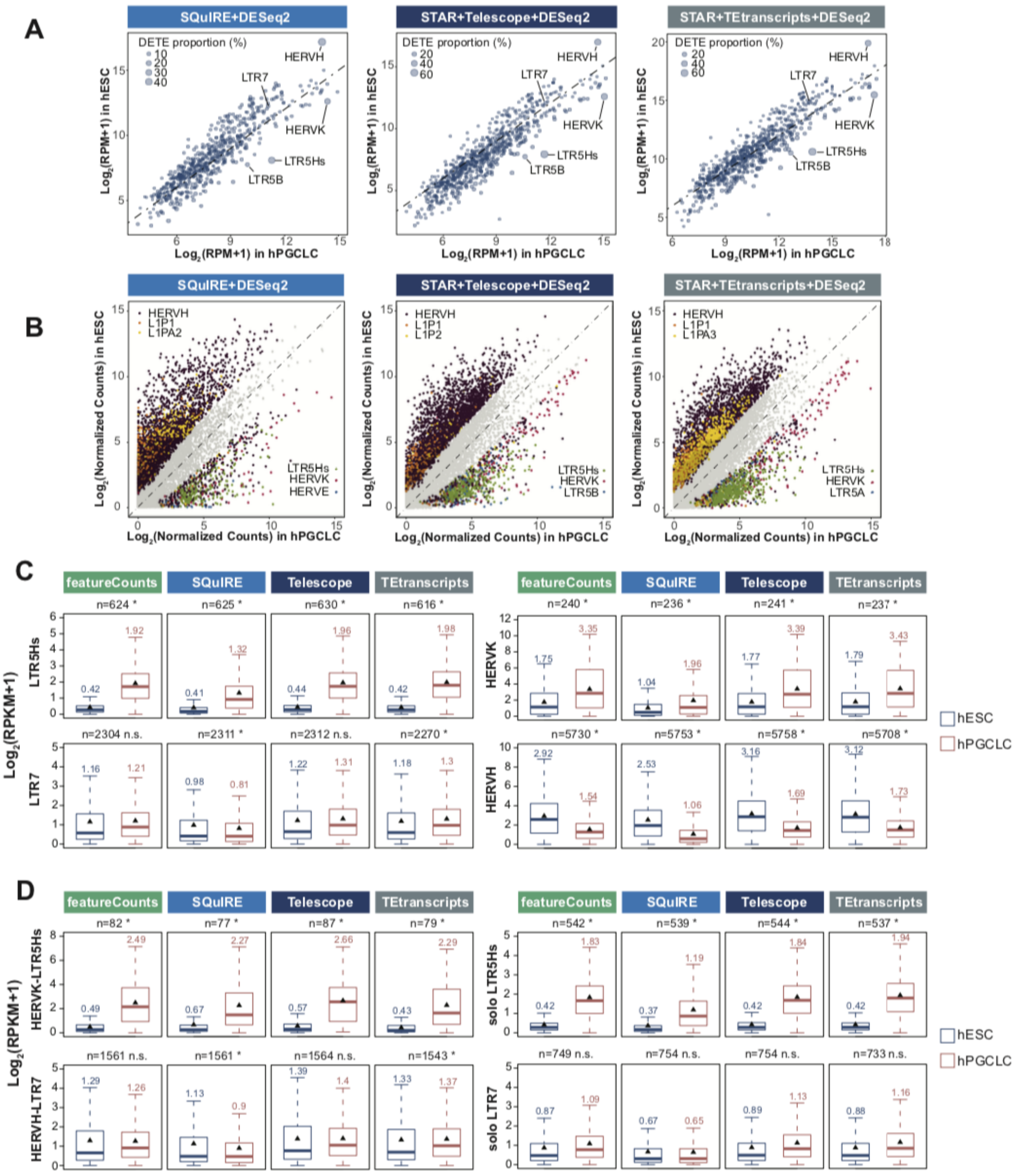

Figure S2. LTR5Hs is specifically expressed in hPGCLCs.

A, B. Scatterplot for aggregated expression level of each TE subfamily (**A**) and individual TE copies belonging to the top three up- or down-regulated DETE subfamilies (**B**) in hESCs and hPGCLCs processed by different methods (left panel: SQuIRE+DESeq2; middle panel: STAR+Telescope+DESeq2; right panel: STAR+TEtranscripts+DESeq2). The size of dots is proportional to DETE copy numbers relative to the total copy number of each TE subfamily. **C.** Boxplot of the expression level of individual TE copies of LTR5Hs, LTR7, HERVK, and HERVH in hESCs and hPGCLCs quantified by different methods. The middle lines represent the median; black triangles represent mean; boxes represent the 25th (bottom) and 75th (top) percentiles; and whisker bars represent the minimum and maximum points within the 1.5× interquartile range. * *p-value* < 0.05, Welch Two Sample t-test; n.s. represents not significant. **D.** Boxplot for the expression level of individual TE copies

of HERVK-LTR5Hs, solo-LTR5Hs, HERVH-LTR7, and solo-LTR7 in hESCs and hPGCLCs quantified by different methods. The middle lines represent the median; black triangles represent mean; boxes represent the 25th (bottom) and 75th (top) percentiles; and whisker bars represent the minimum and maximum points within the 1.5× interquartile range. * *p-value* < 0.05, Welch Two Sample t-test; n.s. represents not significant. Source data underlying Supplementary Figs. 2A, 2B and 2C are provided as a Source Data file.

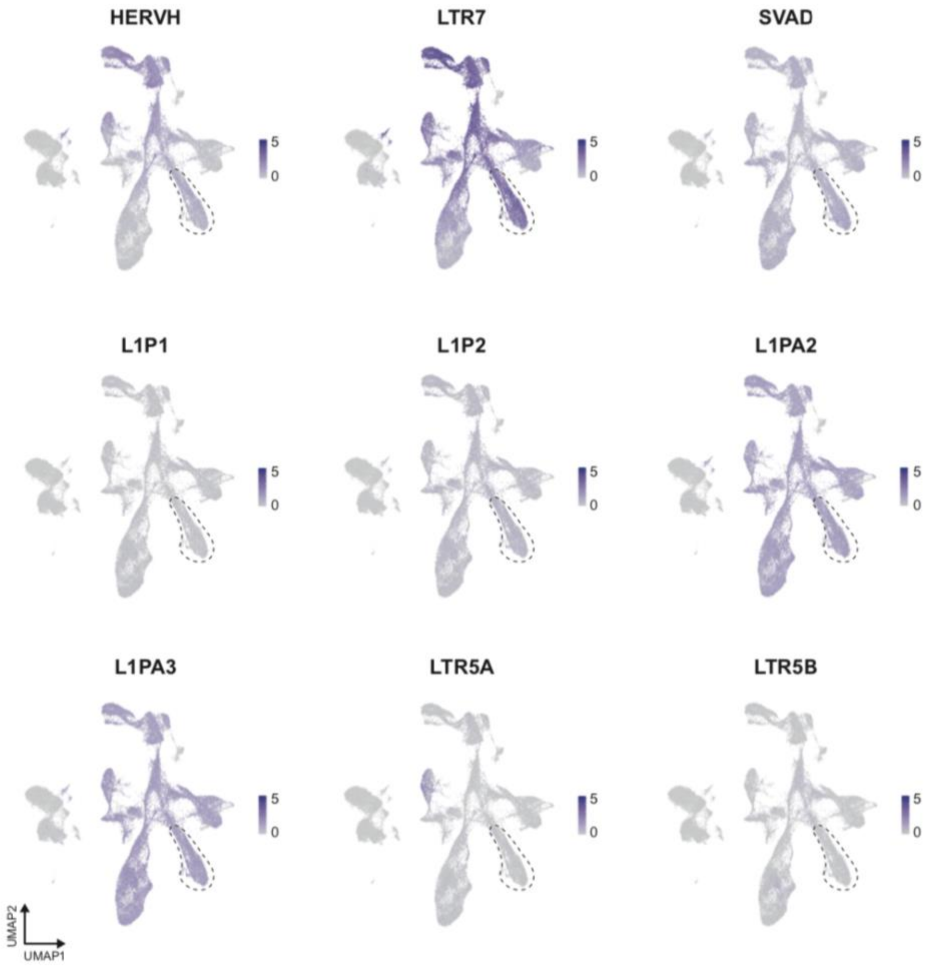

Figure S3. TE expression in hPGCLCs scRNA-seq dataset. UMAP for the expression pattern of representative TE subfamilies during hPGCLC induction at the single cell level. Dotted circle represents the hPGCLC population.

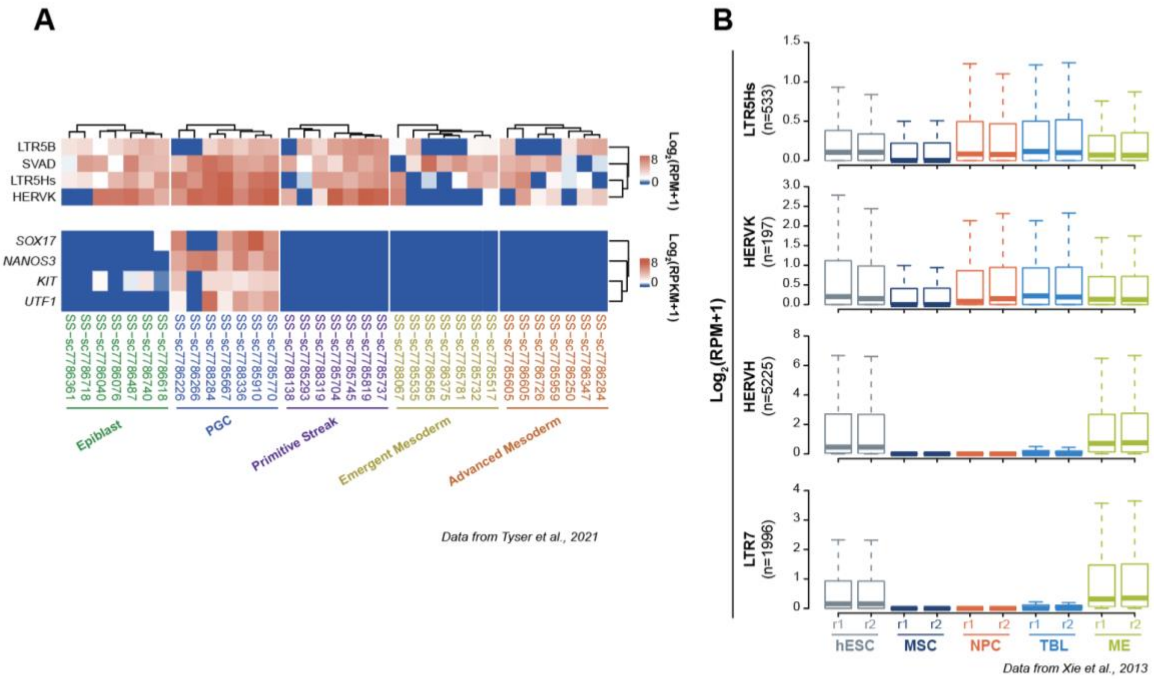

Figure S4. TE expression pattern of *in vivo* hPGCs and *in vitro* hESC multilineage differentiation.

A. Heatmap for the expression levels of different TE subfamilies from the scRNA-seq data in CS7 human embryos[40] across cells belonging to the epiblast, PGCs, primitive streak, emergent mesoderm and advanced mesoderm. Seven individual cells from each lineage are analyzed. **B.** Boxplot showing expression levels of different TE subfamilies from RNA-seq of hESCs following multilineage differentiation *in vitro*[41]. MSC, Mesenchymal Stem Cell; NPC, Neural Progenitor Cell; TBL, Trophoblast-like Cell; ME, Mesendoderm. The middle lines represent the median; boxes represent the 25th (bottom) and 75th (top) percentiles; and whisker bars represent the minimum and maximum points within the 1.5× interquartile range. Source data underlying Supplementary Fig. 4A is provided as a Source Data file.

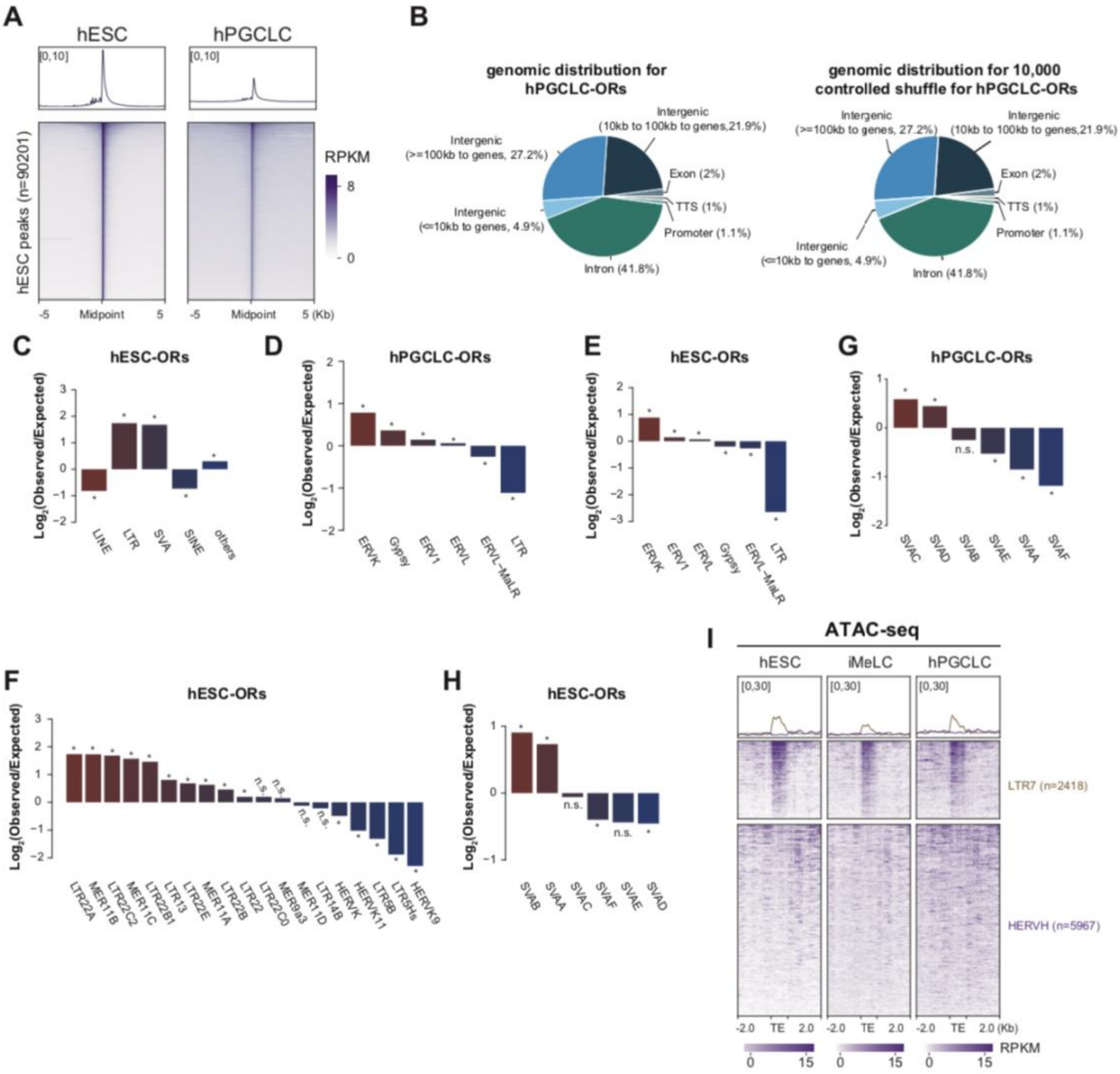

Figure S5. TE subfamilies enriched in hESC-ORs show distinct pattern compared to hPGCLC-ORs.

A. Heatmap and metaplots for ATAC-seq signals over hESC-ORs (n=90201). **B.** Genomic distribution for hPGCLC-ORs (left panel) and random shuffled regions with identical genomic distributions (right panel). **C, E, F, H.** Enrichment of TE classes (**C**), LTR classes (**E**), TE subfamilies in ERVK family (**F**), and TE subfamilies in SVA family (**H**) for hESC-ORs over random shuffled regions with comparable genomic distributions (* *p-value* < 0.05, binomial test; n.s. represents not significant). **D, G.** Enrichment of LTR classes (**D**) and TE subfamilies in SVA family (**G**) for hPGCLC-ORs over random shuffled regions with comparable genomic distributions (* *p-value* < 0.05, binomial test; n.s. represents not significant). **I.** Heatmap and metaplot of ATAC-seq signals over all LTR7 (n=2418) and HERVH (n=5967) copies in hESCs, iMeLCs, and hPGCLCs. Source data underlying Supplementary Figs. 5C-H are provided as a Source Data file.

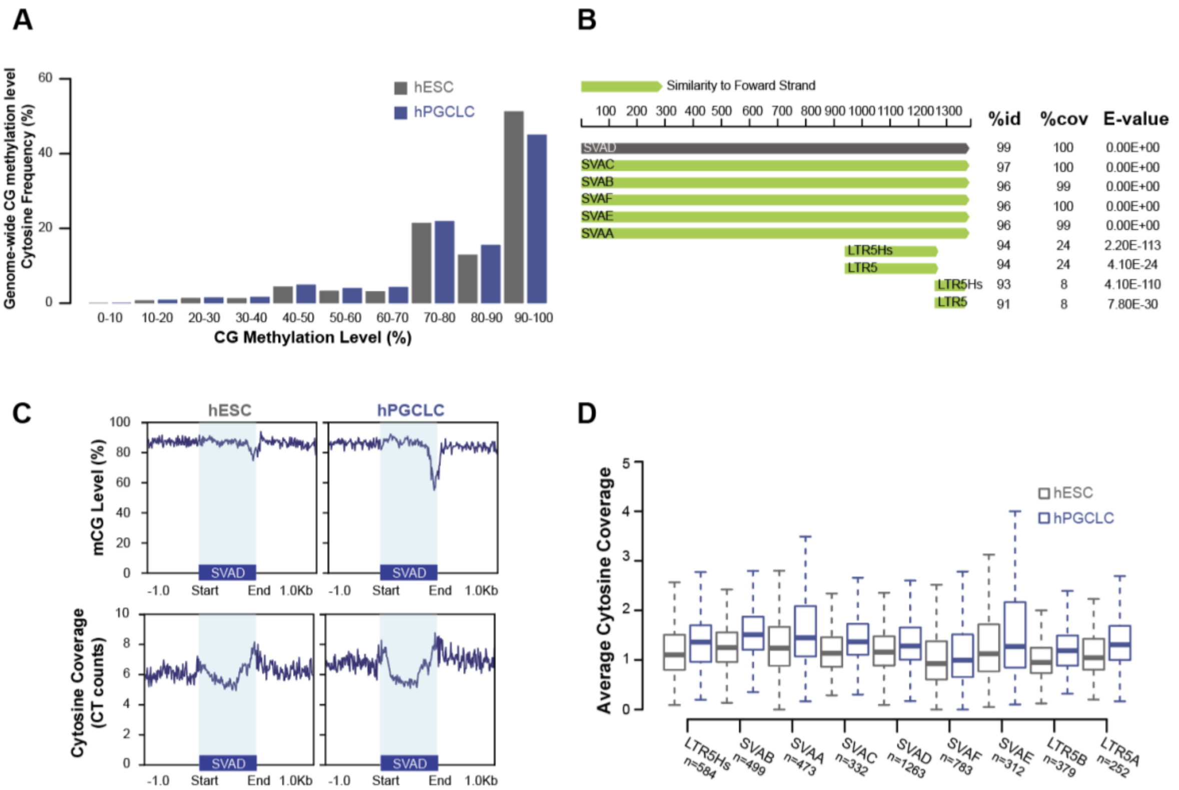

Figure S6. Localized demethylation in hPGCLCs over regions in SVAD sharing sequence similarity with LTR5Hs.

A. Barplot for CG cytosine frequency grouped by CG methylation level in hESCs and hPGCLCs. **B.** The consensus sequence similarity of SVAD and related TE clades from Dfam[45]. Percent identity between the entry consensus sequences (%id), percent shared coverage (%cov) and match e-value (E-value) are displayed on right. **C.** Metaplot of CG methylation level and cytosine coverage over SVAD in hESCs and hPGCLCs. Blue shaded rectangle regions indicate annotated SVAD regions. **D.** Boxplot of cytosine coverage levels over LTR5Hs and related TE clades in hESCs and hPGCLCs. Only TE subfamilies with a copy number greater than 100 are displayed. The middle line represents the median; boxes represent the 25th (bottom) and 75th (top) percentiles; and whisker bars represent the minimum and maximum points within the 1.5× interquartile range. Source data underlying Supplementary Fig. 6A is provided as a Source Data file.

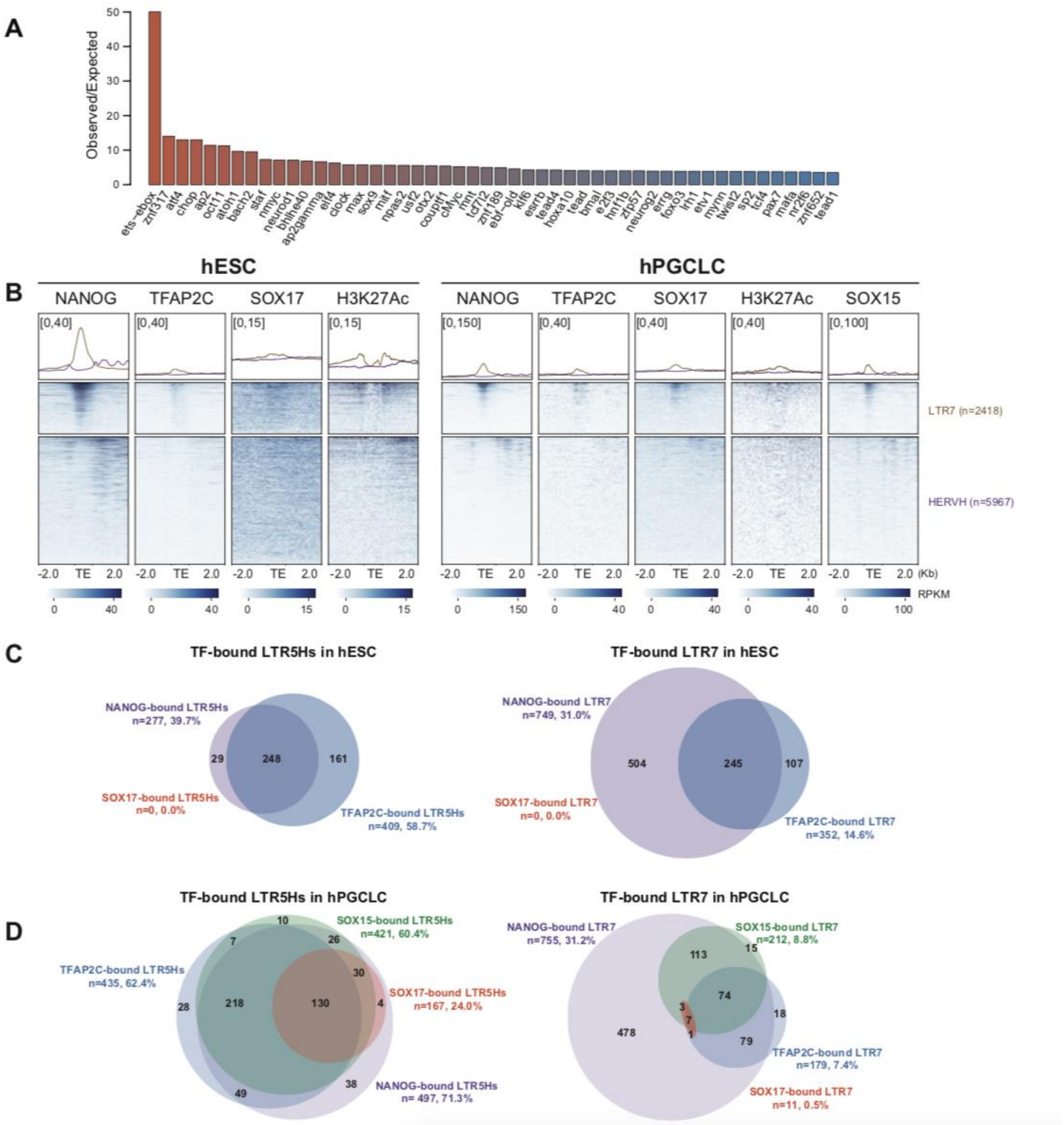

Figure S7. Key PGC transcription factors broadly occupy over LTR5Hs in hPGCLCs.

A. Motif enrichment analysis for LTR5Hs regions overlapped hPGCLC-ORs compared to random shuffled genomic regions. Top 50 TF motifs with highest observed incidence over expected ratios are plotted. **B.** Heatmaps and metaplots of NANOG, TFAP2C, SOX17, and H3K27ac ChIP-seq signals in hESCs and hPGCLCs, and SOX15 CUT&Tag-seq signals in hPGCLCs over all LTR7 (n=2418) and HERVH (n=5967). **C, D.** Venn diagram showing the overlap pattern of TF-bound LTR5Hs and LTR7 regions in hESCs (**C**) and hPGCLCs (**D**). Source data underlying Supplementary Fig. 7A is provided as a Source Data file.

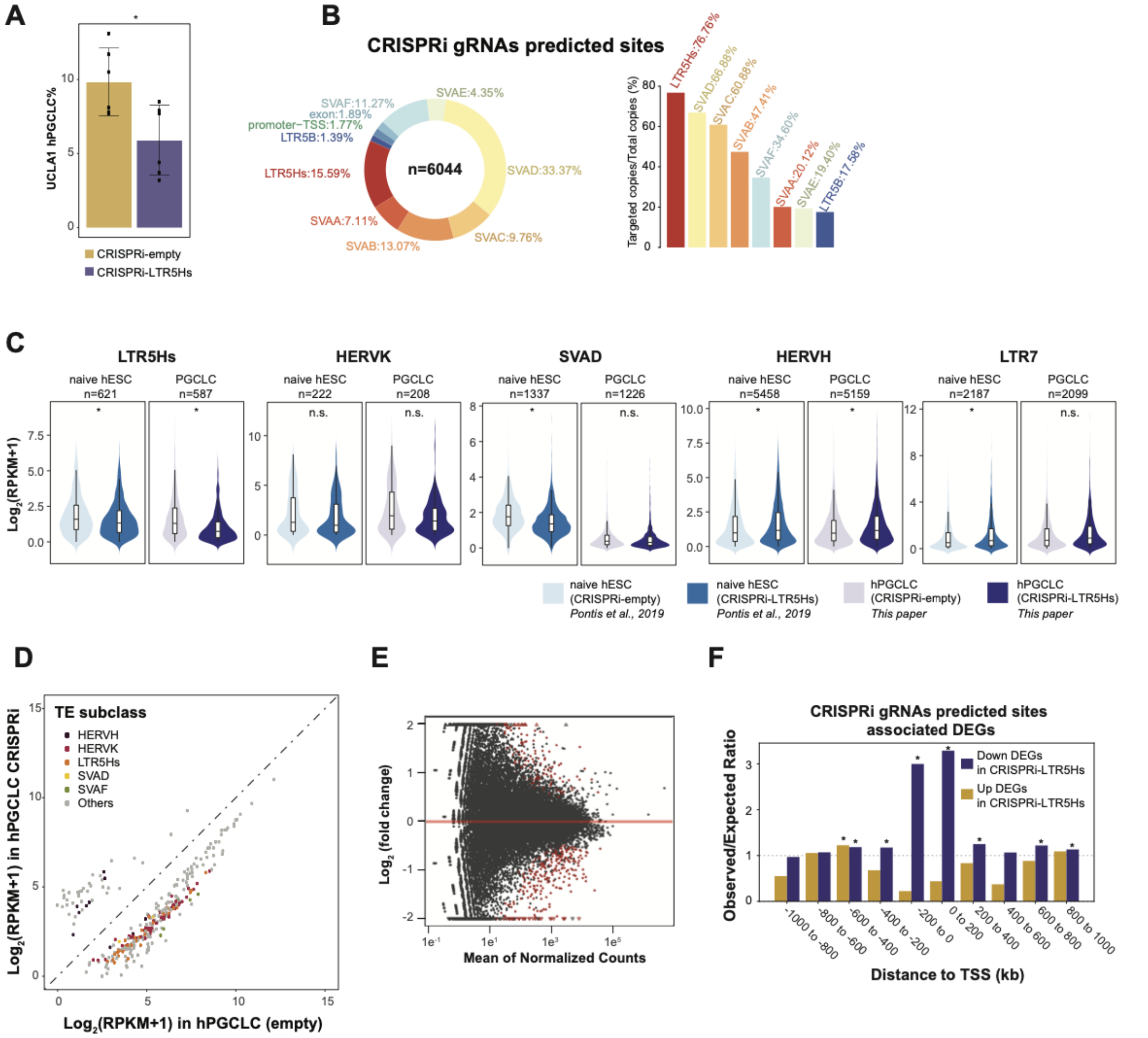

Figure S8. Inactivation of LTR5Hs TEENhancers represses the expression of genes nearby.

A. Barplot showing the percentage of hPGCLCs in CRISPRi-empty relative to CRISPRi-LTR5Hs groups differentiating from UCLA1 hESC lines (biological replicates n=6; *, *p-value*=0.0158; error bars showing mean ± SEM). **B.** Genomic distribution of the predicted sites for LTR5Hs targeting gRNAs (left panel) and the percentage of targeted copy numbers over total copy number for various TE subfamilies (right panel). **C.** Violin and boxplots for the expression levels of various TE subfamilies in CRISPRi-empty vs CRIPRi-LTR5Hs in naïve hESC and hPGCLCs. * *p-value* < 0.05, Welch Two Sample t-test; n.s. represents not significant. The middle lines represent the median; boxes represent the 25th (bottom) and 75th (top) percentiles; and whisker bars represent the minimum and maximum points within the 1.5× interquartile range. **D.** Scatterplot for the expression of individual TE copies belonging representative TE subfamilies. **E.** MA plot showing the distribution of log2 fold changes and the mean of normalized counts for CRISPRi-LTR5Hs and CRIPSRI-empty RNA-seq data. **F.** RAD analysis for the association between LTR5Hs gRNAs predicted sites with CRISPRi-LTR5Hs DEGs. * *p-value* < 0.05,

hypergeometric test. Source data underlying Supplementary Figs. 8A, 8C, and 8F are provided as a Source Data file.

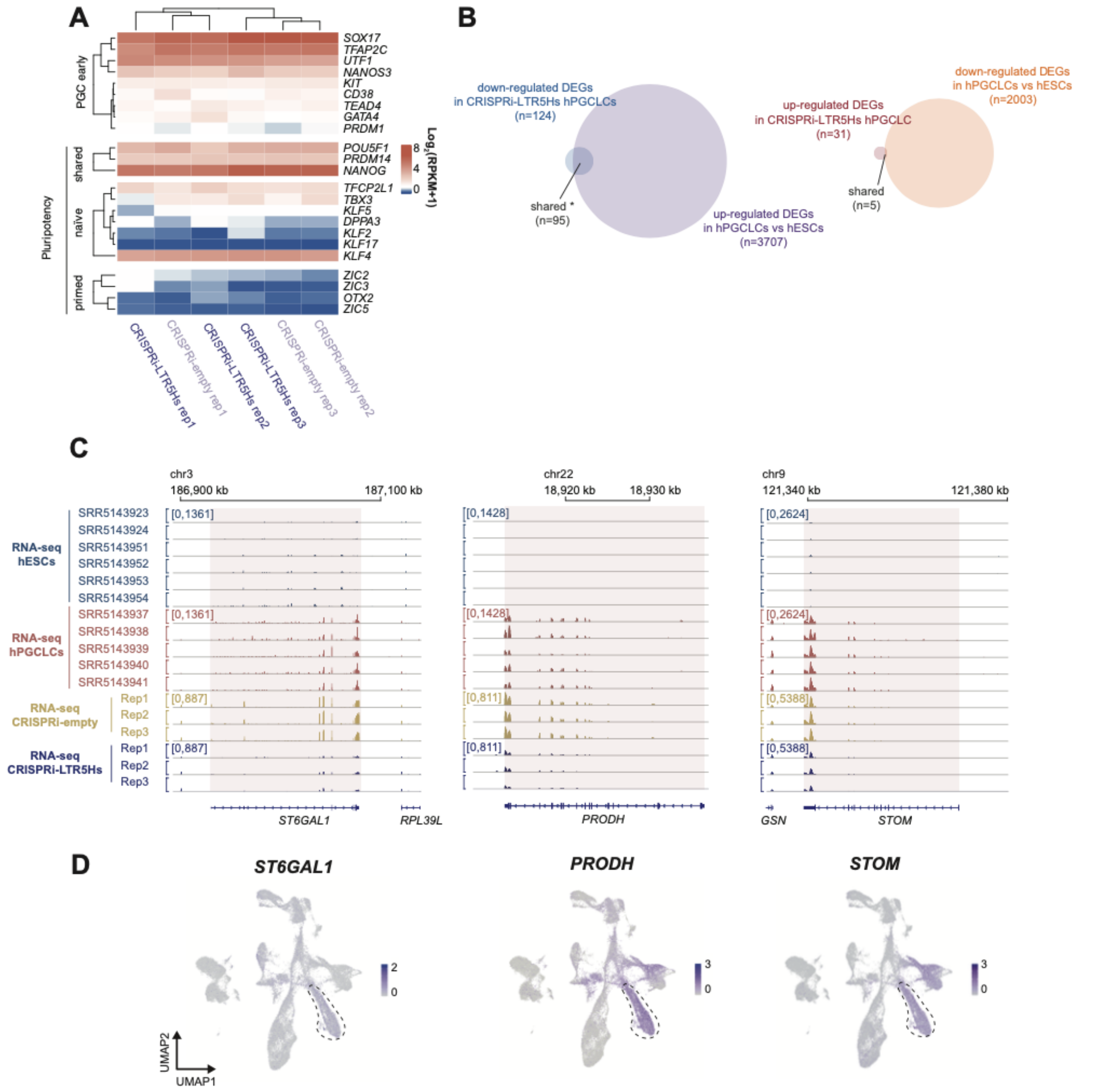

Figure S9. Identification of potential noncanonical LTR5Hs TEENhancer-regulated genes involving in PGC biology.

A. Heatmap for the expression levels of early PGC marker genes and pluripotency genes (shared pluripotency, naïve specific pluripotency and primed specific pluripotency) in the RNA-seq data of CRISPRi-LTR5Hs and CRISPRi-empty hPGCLCs. **B.** Venn diagram for the overlap between down-regulated DEGs in CRISPRi-LTR5Hs hPGCLC and hPGCLC-specific up-regulated DEGs. DEG cutoff is at least 1.5-fold change in expression and a FDR of less than 0.05. * *p-value* < 0.05. **C.** Screenshots of RNA-seq data from hESCs, hPGCLCs, CRIPSRi-empty and CRISPRi-LTR5Hs hPGCLCs over potential target genes of LTR5Hs TEENhancer (*ST6GAL1*, *PRODH*, and *STOM*). **D.** UMAP for the expression pattern of *ST6GAL1*, *PRODH*, and *STOM* during hPGCLC specification at single cell level. Source data underlying Supplementary Figs. 9A and 9B are provided as a Source Data file.
