# Supplementary material for: Human reproduction is regulated by retrotransposons derived from ancient Hominidae-specific viral infections
Source: Nat Commun. 2022 Jan 24;13:463. doi: 10.1038/s41467-022-28105-1 (PMC8786967; doi:10.1038/s41467-022-28105-1)
Supplement: Supplementary file 2 — Reporting Summary [file 41467_2022_28105_MOESM2_ESM.pdf]

nature research

Corresponding author(s): Amander Clark and Wanlu Liu

Last updated by author(s): Nov 7, 2021

Reporting Summary

Nature Research wishes to improve the reproducibility of the work that we publish. This form provides structure for consistency and transparency in reporting. For further information on Nature Research policies, see our Editorial Policies and the Editorial Policy Checklist.

Statistics

For all statistical analyses, confirm that the following items are present in the figure legend, table legend, main text, or Methods section.

n/a	Confirmed	
☐	☒	The exact sample size (*n*) for each experimental group/condition, given as a discrete number and unit of measurement
☐	☒	A statement on whether measurements were taken from distinct samples or whether the same sample was measured repeatedly
☐	☒	The statistical test(s) used AND whether they are one- or two-sided<br>*Only common tests should be described solely by name; describe more complex techniques in the Methods section.*
☒	☐	A description of all covariates tested
☐	☒	A description of any assumptions or corrections, such as tests of normality and adjustment for multiple comparisons
☐	☒	A full description of the statistical parameters including central tendency (e.g. means) or other basic estimates (e.g. regression coefficient) AND variation (e.g. standard deviation) or associated estimates of uncertainty (e.g. confidence intervals)
☐	☒	For null hypothesis testing, the test statistic (e.g. *F*, *t*, *r*) with confidence intervals, effect sizes, degrees of freedom and *P* value noted<br>*Give P values as exact values whenever suitable.*
☒	☐	For Bayesian analysis, information on the choice of priors and Markov chain Monte Carlo settings
☒	☐	For hierarchical and complex designs, identification of the appropriate level for tests and full reporting of outcomes
☒	☐	Estimates of effect sizes (e.g. Cohen's *d*, Pearson's *r*), indicating how they were calculated

Our web collection on statistics for biologists contains articles on many of the points above.

Software and code

Policy information about availability of computer code

Data collection	Flow cytometric data was collected using BD FACSDiva, v8.0.2.<br>RNA-seq and ChIP-seq libraries were subjected to pair-end sequencing on NovaSeq 6000 (Illumina, San Diego).
Data analysis	Software and Algorithms<br>FlowJo FlowJo, v10.6.2, LLC N/A<br>GraphPad Prism, v9.2.0 (283), GraphPad N/A<br>GRCh38 - Genome Assembly N/A<br>FastQC - Quality control of raw reads (Andrews, 2010)<br>v0.11.8<br>STAR - Alignment (Dobin et al., 2012)<br>v2.7.0e<br>SQuIRE - Alignment & transposable element expression quantification (Yang et al., 2019)<br>v0.9.9.92<br>Samtools - Processing post-alignment (Li et al., 2009b)<br>v1.2<br>FeatureCounts - gene and transposable element expression quantification (Liao et al., 2014a)<br>v2.0.0<br>Telescope - transposable element expression quantification (Bendall et al., 2019)<br>v2.0.0<br>TEtranscripts - transposable element expression quantification (Jin et al., 2015)<br>v2.2.1<br>Bedtools multicov - ATAC-seq signal quantification (Quinlan and Hall, 2010)

v2.29.2
Bedtools multiinter & intersect - identifying specific region (Quinlan and Hall, 2010)
v2.29.2
Bedtools shuffle - region shuffling (Quinlan and Hall, 2010)
v2.29.2
10x Genomics Cell Ranger - single cell RNA-seq data alignment and quantification (Zheng et al., 2017)
v3.1.0
Seurat - single cell RNA-seq data analysis and visualization (Stuart et al., 2019)
v3.2.2
R - data processing (R core Team, 2019)
v3.5.1
DESeq2 – DEG calling (Love et al., 2014a)
v1.26.0
MACS2 – Peak calling (Zhang et al., 2008a)
v2.2.7.1
Homer – Region annotation (Heinz et al., 2010b)
v4.7
Deeptools - Bigwig tracks generating; metaplot and heatmap plotting (Ramírez et al., 2014a)
v3.4.3
BSMAP - WGBS data alignment (Xi et al., 2009)
v2.74
wigToBigWig - wiggle file to bigwig file transition (Kent et al., 2010)
v4
DMRcaller - DMR calling (Catoni et al., 2018)
v1.14.2
RAD website application - RAD analysis (Guo et al., 2021)

For manuscripts utilizing custom algorithms or software that are central to the research but not yet described in published literature, software must be made available to editors and reviewers. We strongly encourage code deposition in a community repository (e.g. GitHub). See the Nature Research guidelines for submitting code & software for further information.

Data

Policy information about availability of data

All manuscripts must include a data availability statement. This statement should provide the following information, where applicable:

- Accession codes, unique identifiers, or web links for publicly available datasets
- A list of figures that have associated raw data
- A description of any restrictions on data availability

The accession number for the RNA-seq reported in this paper is GEO: GSE182218.
RNA-seq data (Chen et al., 2018; Xie et al., 2013; Pontis et al., 2019) were collected from GSE93126, GSE16256 and GSE117395.
scRNA-seq data (Chen et al., 2019; Tyser et al., 2020) were collected from GSE140021 and shared by Tyser et al.
ATAC-seq data (Chen et al., 2018) were collected from GSE120648.
WGBS data (Gell et al., 2020) were collected from GSE139115.
ChIP-seq data (Chen et al., 2019; Ji et al., 2015) were collected from GSE140021, GSE69646 and GSE143345.
CUT&Tag-seq data (Wang et al., 2021) were collected from GSE143345.
Custom codes for all the high throughout sequencing analysis are available upon request.

Field-specific reporting

Please select the one below that is the best fit for your research. If you are not sure, read the appropriate sections before making your selection.

☒ Life sciences ☐ Behavioural & social sciences ☐ Ecological, evolutionary & environmental sciences

For a reference copy of the document with all sections, see nature.com/documents/nr-reporting-summary-flat.pdf

Life sciences study design

All studies must disclose on these points even when the disclosure is negative.

Sample size	No effect sizes were pre-specified.
Data exclusions	No data were excluded from this work.
Replication	For CRISPRi experiments in UCLA2: hPGCLC differentiation was performed n=3 independent times for each group. For UCLA1: hPGCLC differentiation was performed n=6 independent times for each group. RNA-Seq for CRISPRi experiments in UCLA2: we performed RNA-Seq in n=3 biological replicates<br>For ChIP-seq of NANOG and SOX17: We performed ChIP-seq in n=2 biological replicates for NANOG ChIP-seq in both UCLA2 hESCs and induced hPGCLCs; we performed ChIP-seq in n=2 biological replicates for SOX17 ChIP-seq in both UCLA2 hESCs and induced hPGCLCs.

Randomization	For CRISPRi experiments, no selective process was used to assign experimental groups, instead cells (UCLA1 or UCLA2) were randomly split into treatment or control groups at the beginning of the experiment.<br>For all ChIP-Seq experiments, randomization does not apply because there were no treatment groups.
Blinding	NA. Blinding was not necessary as quantitative analysis methods were performed in batch.

Reporting for specific materials, systems and methods

We require information from authors about some types of materials, experimental systems and methods used in many studies. Here, indicate whether each material, system or method listed is relevant to your study. If you are not sure if a list item applies to your research, read the appropriate section before selecting a response.

Materials & experimental systems

n/a	Involved in the study	
☐	☒	Antibodies
☐	☒	Eukaryotic cell lines
☒	☐	Palaeontology and archaeology
☒	☐	Animals and other organisms
☒	☐	Human research participants
☒	☐	Clinical data
☒	☐	Dual use research of concern

Methods

n/a	Involved in the study	
☐	☒	ChIP-seq
☐	☒	Flow cytometry
☒	☐	MRI-based neuroimaging

Antibodies

Antibodies used	BV421 conjugated anti-human/mouse CD49f (ITGA6) at 1/80; BioLegend; Cat#313624; RRID: AB_2562244; Lot#B274314<br>APC-conjugated anti-human CD326 (EPCAM) at 1/80; BioLegend ; Cat#324208; RRID: AB_756082; Lot#B284158<br>anti-NANOG antibody (Cat#AF1997, R and D Systems), 1.2ug; anti-SOX17 antibody (Cat#AF1924, R and D Systems), 1.6ug
Validation	For BV421 conjugated anti-human/mouse CD49f (ITGA6) and APC-conjugated anti-human CD326 (EPCAM), verification from BioLegend website: Each lot of this antibody is quality control tested by immunofluorescent staining with flow cytometric analysis.<br>For anti-NANOG and anti-SOX17 antibody, ChIP examples are also shown on the R and D Systems website.

Eukaryotic cell lines

Policy information about cell lines

Cell line source(s)	UCLA1 and UCLA2 are human embryonic stem cell (hESC) lines. The lines were generated at UCLA in 2009 by the UCLA Pluripotent Stem Cell Core Facility. Each cell line was generated from a single human embryo consented for research. Following derivation the hESC lines are de-identified, and provided to investigators with all links and identifiers removed. SNP/CNV analysis using the Affymetrix Genome-wide Human SNP Array 6.0 was used for authentication of each hESC line before distribution. The research performed in this study is not considered human subjects research because the cells are provided from the UCLA core facility to UCLA investigators de-identified.<br>HEK293T cells are purchased directly from ATCC (Cat # CRL-3216). Authentication involved certification by ATCC (Manassas, Virginia) prior to purchase.
Authentication	SNP/CNV Analysis was used for authentication
Mycoplasma contamination	Mycoplasma testing is performed on a routine basis using ELISA and the cell line is negative.
Commonly misidentified lines (See ICLAC register)	None

ChIP-seq

Data deposition

☒ Confirm that both raw and final processed data have been deposited in a public database such as GEO.

☒ Confirm that you have deposited or provided access to graph files (e.g. BED files) for the called peaks.

Data access links<br>*May remain private before publication.*	GSE182218 [https://www.ncbi.nlm.nih.gov/geo/query/acc.cgi?acc=GSE182218]
Files in database submission	GSM5525241 NANOG input hESC rep1<br>GSM5525242 NANOG ChIP hESC rep1<br>GSM5525243 NANOG input hESC rep2<br>GSM5525244 NANOG ChIP hESC rep2

GSM5525245 NANOG input hPGCLC rep1
GSM5525246 NANOG ChIP hPGCLC rep1
GSM5525247 NANOG input hPGCLC rep2
GSM5525248 NANOG ChIP hPGCLC rep2
GSM5525249 SOX17 input hESC rep1
GSM5525250 SOX17 ChIP hESC rep1
GSM5525251 SOX17 input hESC rep2
GSM5525252 SOX17 ChIP hESC rep2
GSM5525253 SOX17 input hPGCLC rep1
GSM5525254 SOX17 ChIP hPGCLC rep1
GSM5525255 SOX17 input hPGCLC rep2
GSM5525256 SOX17 ChIP hPGCLC rep2

Genome browser session (e.g. UCSC): not applicable

Methodology

Replicates: We performed ChIP-seq in n=2 biological replicates for NANOG ChIP-seq in both UCLA2 hESCs and induced hPGCLCs; we performed ChIP-seq in n=2 biological replicates for SOX17 ChIP-seq in both UCLA2 hESCs and induced hPGCLCs.

Sequencing depth: NANOG ChIP-seq in UCLA2 hESCs: Total number of reads is 77991139. Uniquely mapped reads is 62524610. Valid reads is 46225005 (reads after removing PCR duplicate). Read is paired end 100 bp sequenced.
NANOG ChIP-seq in UCLA2 induced hPGCLCs: Total number of reads is 58338936. Uniquely mapped reads is 50835858. Valid reads is 39374442 (reads after removing PCR duplicate). Read is paired end 100 bp sequenced.

SOX17 ChIP-seq in UCLA2 hESCs: Total number of reads is 61168179. Uniquely mapped reads is 34019904. Valid reads is 23125706 (reads after removing PCR duplicate). Read is paired end 100 bp sequenced.
SOX17 ChIP-seq in UCLA2 induced hPGCLCs: Total number of reads is 90840663. Uniquely mapped reads is 47355137. Valid reads is 23602582 (reads after removing PCR duplicate). Read is paired end 100 bp sequenced.

Antibodies: anti-NANOG antibody (Cat#AF1997, R and D Systems), 1.2ug
anti-SOX17 antibody (Cat#AF1924, R and D Systems), 1.6ug

Peak calling parameters: ChIP-seq peaks were defined using the MACS2 v2.2.7.1 callpeaks function by setting ChIP file as treatment and input file as control.

Data quality: Quality control was performed by FastQC v0.11.8.

Software: FastQC v0.11.8 is used for quality control. STAR v2.7.0e is used for alignment. Samtools v1.9 is used for processing post-alignment. Deeptools v3.4.3 is used for Bigwig tracks generating and plotting. MACS2 v2.2.7.1 is used for peak calling. Homer v4.7 is used for motif annotation.

Flow Cytometry

Plots

Confirm that:

- [x] The axis labels state the marker and fluorochrome used (e.g. CD4-FITC).
- [x] The axis scales are clearly visible. Include numbers along axes only for bottom left plot of group (a 'group' is an analysis of identical markers).
- [x] All plots are contour plots with outliers or pseudocolor plots.
- [x] A numerical value for number of cells or percentage (with statistics) is provided.

Methodology

Sample preparation: hPGCLC aggregates were dissociated with 0.05% Trypsin-EDTA 0.05% trypsin-EDTA (Thermo Fisher Scientific, 25300120) for 10 min at 37 C. The dissociated cells were then stained with conjugated antibodies, washed with FACS buffer (1% BSA in PBS) and resuspended in FACS buffer with 7-AAD (BD PharMingen, 559925) as viability dye. The single cell suspension was sorted for further experiments.

Instrument: BD FACS ARIA

Software: For collection: BD FACSDiva v8.0.2; for data analysis: FlowJo_v10.6.2_CL.

Cell population abundance: Post-sort efficiency is more than 90% for all samples.

Gating strategy: 1. FSC/SSC gating to remove debris; 2. single cell gating to select for single cells; 3. 7-AAD negative cells for live cells; 4. select for BV421 and APC double positive cells as hPGCLC.

- [x] Tick this box to confirm that a figure exemplifying the gating strategy is provided in the Supplementary Information.
