# Supplementary material for: Human reproduction is regulated by retrotransposons derived from ancient Hominidae-specific viral infections
Source: Nat Commun. 2022 Jan 24;13:463. doi: 10.1038/s41467-022-28105-1 (PMC8786967; doi:10.1038/s41467-022-28105-1)
Supplement: Supplementary file 3 — Description of Additional Supplementary Files [file 41467_2022_28105_MOESM3_ESM.docx]

**Description of Additional Supplementary Files**

**Title: Supplementary Data 1**

Description: Information of samples used in this article. RNA-seq, scRNA-seq, ATAC-seq, ChIP-seq, CUT&Tag-seq and WGBS data information.

**Title: Supplementary Data 2**

Description: List of up- or down-regulated DETEs in hPGCLCs compared with hESCs from featureCounts quantification method. TE quntification was performed by featureCounts v2.0.0 (Liao et al., 2014). DETEs were processed using DESeq2 v1.26.0 (Love et al., 2014b). Only TE with RPKM mean in either control or treatment group greater than 1 were kept for further analysis. DETEs were obtained with at least 4-fold change and FDR less than 0.05. The data was used to generate Fig. 1C-D, F-G.

**Title: Supplementary Data 3**

Description: List of up- or down-regulated DETEs in hPGCLCs compared with hESCs from SQuIRE quantification method. TE quntification was performed by SQuIRE v.0.9.9.92 (Yang et al., 2019). DETEs were processed using DESeq2 v1.26.0 (Love et al., 2014b). Only TE with RPKM mean in either control or treatment group greater than 1 were kept for further analysis. DETEs were obtained with at least 4-fold change and FDR less than 0.05. The data was used to generate Supplementary Fig. 1B-C, 2A-B.

**Title: Supplementary Data 4**

Description: List of up- or down-regulated DETEs in hPGCLCs compared with hESCs from Telescope quantification method. TE quntification was performed by Telescope v.2.0.0 (Bendall et al., 2019). DETEs were processed using DESeq2 v1.26.0 (Love et al., 2014b). Only TE with RPKM mean in either control or treatment group greater than 1 were kept for further analysis. DETEs were obtained with at least 4-fold change and FDR less than 0.05. The data was used to generate Supplementary Fig. 1B-C, 2A-B.

**Title: Supplementary Data 5**

Description: List of up- or down-regulated DETEs in hPGCLCs compared with hESCs from TEtranscripts quantification method. TE quntification was performed by TEtranscripts v.2.2.1 (Jin et al., 2015). DETEs were processed using DESeq2 v1.26.0 (Love et al., 2014b). Only TE with RPKM mean in either control or treatment group greater than 1 were kept for further analysis. DETEs were obtained with at least 4-fold change and FDR less than 0.05. The data was used to generate Supplementary Fig. 1B-C, 2A-B.

**Title: Supplementary Data 6**

Description: List of hPGCLC-ORs and hESC-ORs. ATAC-seq peak specific open region (OR) coordinates for hPGCLC-ORs (n=31276) and hESC-ORs (n=90201). The data was used to generate Fig. 2A-C and Supplementary Fig. 5A-H .

**Title: Supplementary Data 7**

Description: List of DMRs in hPGCLCs compared with hESCs. DMR were defined using DMRcaller v1.14.2 (Catoni et al., 2018) over GRCh38 whole genome using 200bp as DMR bin size. Only bins with at least four CG sites and each CG sites should be covered by at least three reads were kept for further analysis. Minimal CG methylation difference of 0.2 and FDR less than 0.05 were applied to define DMRs. Bins defined as DMR and within 100bp gap were merged. The data was used to generate Fig. 3A-B.

**Title: Supplementary Data 8**

Description: List of TF bound LTR5Hs and LTR7. The TF bound pattern over LTR5Hs (n=697) and LTR7 (n=2418) copies. The data was used to generate Supplementary Fig. 7C-D.

**Title: Supplementary Data 9**

Description: List of up- or down-regulated DETEs in CRISPRi-LTR5Hs compared with CRISPRi-empty. TE quntification was performed by featureCounts v2.0.0 (Liao et al., 2014). DETEs were processed using DESeq2 v1.26.0 (Love et al., 2014b). Only TE with RPKM mean in either control or treatment group greater than 1 were kept for further analysis. DETEs were obtained with at least 4-fold change and FDR less than 0.05. The data was used to generate Fig. 5D-E.

**Title: Supplementary Data 10**

Description: List of CRISPRi gRNAs predicted sites. Homer v4.7 (Heinz et al., 2010a) was used to search the targeting sites for LTR5Hs gRNA plus PAM NGG sequence (CTCCCTAATCTCAAGTACCCNGG, TGTTTCAGAGAGCACGGGGTNGG) with less than 3 mismatches. The target sites were annotated using gene and TE annotation and then categorized into either promoter, exonic, TE, intronic, or intergenic sites. If one target site was annotated with multiple categories, only one category would be retained with priority order of promoter, exon, TE, intron, and intergenic sites. The data was used to generate Supplementary Fig. 8B.

**Title: Supplementary Data 11**

Description: List of up- or down-regulated DEGs in CRISPRi-LTR5Hs compared with CRISPRi-empty. Gene quntification was performed by featureCounts v2.0.0 (Liao et al., 2014). DEGs were processed using DESeq2 v1.26.0 (Love et al., 2014b). Only gene with RPKM mean in either control or treatment group greater than 1 were kept for further analysis. DEGs were obtained with at least 1.5-fold change and FDR less than 0.05. The data was used to generate Fig. 5F and Supplementary Fig. 9B.

**Title: Supplementary Data 12**

Description: List of LTR5Hs associated up- or down-regulated DEGs in CRISPRi-LTR5Hs compared with CRISPRi-empty. RAD analysis was performed by website application from (Guo et al., 2021). For RAD analysis of LTR5Hs associated DEGs, up- and down-regulated DEGs in CRISPRi-LTR5Hs were input as DEGs lists; LTR5Hs bed file or randomly shuffled LTR5Hs bed file by bedtools v2.29.2 shuffle function (Quinlan and Hall, 2010) were input as Genomic Regions of Interest (gROI) file. For submit options, “GRCh38” was chose for reference genome, “1000, 800, 600, 400, 200, 0 kb” was input as customized peak extend distance, “hypergeometric test” was chose for statistical test. The data was used to generate Fig. 5G.

**Title: Supplementary Data 13**

Description: List of CRISPRi gRNAs predicted sites associated up- or down-regulated DEGs in CRISPRi-LTR5Hs compared with CRISPRi-empty. RAD analysis was performed by website application from (Guo et al., 2021). For RAD analysis of CRISPRi gRNAs predicted sites associated DEGs, up- and down-regulated DEGs in CRISPRi-LTR5Hs were input as DEGs lists; bed file of CRISPRi gRNAs predicted sites was input as gROI file. For submit options, “GRCh38” was chose for reference genome, “1000, 800, 600, 400, 200, 0 kb” was input as customized peak extend distance, “hypergeometric test” was chose for statistical test. The data was used to generate Supplementary Fig. 8F.

**Title: Supplementary Data 14**

Description: List of up- or down-regulated DEGs in hPGCLCs compared with hESCs. Gene quntification was performed by featureCounts v2.0.0 (Liao et al., 2014). DEGs were processed using DESeq2 v1.26.0 (Love et al., 2014b). Only gene with RPKM mean in either control or treatment group greater than 1 were kept for further analysis. DEGs were obtained with at least 1.5-fold change and FDR less than 0.05. The data was used to generate Supplementary Fig. 9B.
